# Novel *Lactobacillus reuteri* HI120 Affects Lipid Metabolism in C57BL/6 Obese Mice

**DOI:** 10.3389/fvets.2020.560241

**Published:** 2020-10-14

**Authors:** Ye Sun, Yanqing Tang, Xufeng Hou, Hesong Wang, Liuying Huang, Junjie Wen, Hongxin Niu, Weisen Zeng, Yang Bai

**Affiliations:** ^1^Guangdong Provincial Key Laboratory of Gastroenterology, Department of Gastroenterology, Institute of Gastroenterology of Guangdong Province, Nanfang Hospital, Southern Medical University, Guangzhou, China; ^2^Department of General Medicine, Zhujiang Hospital, Southern Medical University, Guangzhou, China; ^3^Department of Cell Biology, School of Basic Medicine, Southern Medical University, Guangzhou, China; ^4^Guangzhou Weisengene Biological Technology Co., Ltd, Guangzhou, China

**Keywords:** probiotics, obesity, cholesterol, lipid metabolism, conjugated linoleic acid, *Lactobacillus reuteri*

## Abstract

Intestinal probiotics are a primary focus area of current medical research. Probiotics such as bifidobacteria and lactobacilli can positively impact obesity and other metabolic diseases by directly or indirectly affecting lipid metabolism. However, the precise mechanisms of these effects remain unclear. In our previous work, the novel strain *Lactobacillus reuteri* HI120 was isolated and identified. HI120 expresses high levels of linoleic isomerase, resulting in the production of large amounts of conjugated linoleic acid (CLA) when mixed with linoleic acid (LA). As HI120 can efficiently transform LA into CLA, the effect of HI120 on the lipid metabolism in C57BL/6 obese mice was studied and the underlying molecular mechanism was explored *in vitro*. The results revealed no significant change in the diet, body weight, and serum triglyceride levels in mice. However, serum cholesterol levels were significantly decreased. The underlying mechanism may involve a CLA-mediated reduction in the gene expression levels of *NPC1L1, SREBP-2*, and *HMG-CR*, resulting in reduced cholesterol synthesis and absorption. Thus, HI120 can be developed as a potential probiotic formulation. After oral administration, LA from certain food sources can be converted into CLA in the human intestine to contribute to the prevention and treatment of obesity and hyperlipidemia.

## Introduction

Obesity can cause a series of conditions such as hyperlipidemia, fatty liver, atherosclerosis, hypertension, type 2 diabetes, and even malignant tumors, all of which are harmful to human health (1–4).

Gut microbes can affect obesity, insulin resistance, diabetes, and metabolic syndrome, among other conditions (5). Gut microbes play important roles in metabolic diseases and can directly or indirectly affect lipid metabolism. However, the exact mechanism of these interactions remains unclear (6). As early as 1963, probiotics were found to have a cholesterol-lowering effect in humans (7, 8). The cholesterol level in the plasma of a high-fat-induced atherosclerosis animal model was decreased by administration of a lactobacillus-rich diet (9). Two strains of *Lactobacillus plantarum*, ATG-K2 and ATG-K6, can significantly reduce body weight, liver fat accumulation, and related gene expression in non-alcoholic fatty liver disease (NAFLD) rat models generated by a high-fat/high-fructose diet (10). Thus, certain probiotics may be useful as biological agents for preventing and controlling lipid metabolism-related diseases.

Certain lactobacillus strains, such as *Lactobacillus reuteri, L. plantarum*, and *Lactobacillus acidophilus*, contain linoleic acid isomerase (LAI), which can isomerize linoleic acid (LA) into two isomers of conjugated linoleic acid (CLA) containing conjugated double bonds (11). *Lactobacillus reuteri* has been reported to exist in the intestines of almost all vertebrates and mammals. Because of its beneficial effects, it is considered as a probiotic. In our previous work, we isolated a *L. reuteri* strain expressing high levels of LAI from the feces of healthy children and named this strain as HI120. Mixing *L. reuteri* HI120 with LA *in vitro* resulted in the generation of a large amount of CLA, as revealed by mass spectrometry. In this study, we performed sequencing and mass spectrometry analysis of HI120. In 2008, the FDA issued a food license for the use of CLA as a food additive (12). CLA shows effects against cancer, obesity, and atherosclerosis (13) as well as potent functions in weight and fat reduction. Sprague-Dawley rats fed CLA displayed lower body fat, weight, triglyceride, and cholesterol levels (14). The body weight of *ob*/*ob* mice was reduced after feeding of c9, t11-CLA (15). Furthermore, t10, c12-CLA reduced triglycerides and cholesterol levels in the liver of obese rats (16). Daily supplementation of 0.7–1.4 g CLA in patients with hyperlipidemia significantly reduced the concentrations of serum low-density lipoprotein-cholesterol, very low-density lipoprotein-cholesterol, and triglycerides (17). When 3.0% CLA was added to the diet of mice, the concentration of total cholesterol in the liver decreased by 41% (18). Based on these results, we fed C57BL/6 obese mice with HI120 and sunflower seed oil containing 60% LA to simulate the everyday human diet and observe whether any fat- and weight-reducing effects occurred. *Lactobacillus reuter*i DSM20016 is the standard strain of *L. reuteri*, which is often used as a control with other bacteria (19). In this study, *L. reuter*i DSM20016 was used as the control to compare the weight loss and lipid-lowering effects of HI120. Atorvastatin is a commonly used lipid-lowering drug in the clinic (20, 21). We also used atorvastatin in the positive control group to understand the lipid-lowering effect of HI120. *NPC1L1, SREBP-2, HMG-CR, CYP7A1, FXR*, and *LXR* (22–27) are important genes involved in cholesterol synthesis, absorption, and excretion. We examined these genes *in vitro* and *in vivo* to explore the molecular mechanism of HI120 in lowering cholesterol.

## Materials and Methods

### Ethics Statement

Animal experiments were performed in strict accordance with the National Institutes of Health guidelines and were approved by the Institutional Laboratory Animal Care and Use Committee of the South Medical University at Guangzhou (Approval No. L2017104, Guangzhou, Guangdong province, China).

### Bacterial Strains, Animals, Cell Lines, and Culture Conditions

*Lactobacillus reuteri* HI120 is preserved at the culture preservation center of Guangdong Institute of Microbiology (DEPOSIT No.: GDWSD0600119, China). *Lactobacillus reuteri* DSM20016 (Deutsche Sammlung von Mikroorganismem und Zellkulturen'scollection, Braunschweig, Germany) was used in this study. Both species were verified by PCR amplification.

Male C57BL/6 mice (6–8-week-old) were obtained from the Southern Medical University animal facility (China) and reared under specific pathogen-free conditions. All animals were housed under controlled conditions (temperature 20 ± 2°C, humidity 55 ± 5%, 12/12 h light/dark cycle; 08:00–20:00 h) with free access to tap water and diet, and maintained in groups in cages with cottonwood sawdust pads. All mice were fed either a normal diet [32% (w/w) protein, 5% fat, 2% fiber, 1.8% calcium, 1.2% phosphorus, and 58% nitrogen free extract] or a high-fat diet [10% (w/w) lard, 1% cholesterol, 0.2% sodium cholate, and 88.8% normal diet].

The human colorectal adenocarcinoma cell line Caco-2 and hepatoma cell line HepG2 were provided by Southern Medical University (Guangzhou, China) and routinely cultured in RPMI 1640 medium (Gibco, Grand Island, NY, USA) and DMEM high-glucose culture medium (Gibco), respectively, supplemented with 10% heat-inactivated fetal bovine serum (PAN, Aidenbach, Germany), penicillin (100 U/mL), and streptomycin (100 ng/mL). Cell cultures were maintained in an incubator at 37°C under 95% (v/v) humidified air and 5% (v/v) CO_2_.

### Sequencing Identification of *L. reuteri* HI120 and Detection of Its LA–CLA Conversion Rate

HI120 was cultured and lysozyme was added to lyse the bacteria for genomic DNA extraction. 16S rRNA universal primers were used, and genomic DNA was used as template for PCR. Using the NCBI database, the 16S rRNA gene sequence of HI120 was compared with that of various *L. reuteri* strains using BLAST software. The LAI amino acid sequence of HI120 was also compared to that of other strains.

To detect the transformation rate of LA to CLA by HI120, several experimental groups were established. LA group: 1 mg/mL LA and 1 mg/mL Tween 80 MRS medium incubated at 37°C on a shaking table at 120 rpm for 48 h. DSM20016 + LA group: DSM20016 inoculated at a 1:100 ratio and cultured on a shaking table at 120 rpm and 37°C for 24 h. Subsequently, DSM20016 was inoculated at 5% v/v into MRS medium containing 1 mg/mL LA and 1 mg/mL Tween 80, followed by culture at 120 rpm and 37°C on a shaking table for 48 h. HI120 + LA group: HI120 inoculated at a 1:100 ratio, cultured on a shaking table at 120 rpm and 37°C for 24 h, and subsequently inoculated at 5% v/v into MRS medium containing 1 mg/mL LA and 1 mg/mL Tween 80, followed by culture on a shaking table at 120 rpm and 37°C for 48 h. The procedure for each group was carried out at least three times in parallel. The bacterial solution mixture was centrifuged at 5,000 rpm for 5 min, and the supernatant was collected. A volume of 1 mL culture medium + 2 mL isopropanol + 1.5 mL *n*-hexane was added for extraction for 3 min followed by centrifugation of the sample at 6,000 rpm for 5 min. The upper layer of *n*-hexane was absorbed, dried with anhydrous sodium sulfate, and placed for stratification. For gas chromatography-mass spectrometry (Agilent, Santa Clara, CA, USA) detection, the following parameters were used: sample inlet 280°C, constant temperature at 50°C for 2 min, and then the temperature was increased at 15°C/min up to 250°C. A constant temperature was maintained for 10 min with a sample volume of 0.2 μL. The external standard method was used for qualitative and quantitative detection, with c9, t11-CLA standard, t10, c12-CLA standard, and LA standard as standard samples and *n*-hexane as solvent.

### Preparation of Bacterial Powder and Detection of Its Concentration

Powdered bacterial protective agent (100 mL deionized water with 4.5 g dissolved gelatin + 0.9 g sodium chloride + 3 g fructose + 3 g starch) was added to the high-concentration HI120/DSM20016 bacterial solution, and the mixture was suspended and precipitated again. This mixture was dried using a powder-spraying machine (drying conditions: rotation speed 15 rpm, drying temperature 115°C, fan frequency 50 Hz). The dry powder was collected, packed separately, marked, and dried at 4°C. Next, 100 mg of HI120 and DSM20016 bacterial powder was used to prepare 10 mL of solution. A volume of 100 μL of solution was collected and serially diluted by 10-fold at 10^7^, 10^8^, 10^9^, and 10^10^. This diluted solution (100 μL) was applied to the solid medium. The mixtures were then labeled and cultured for 48 h at 37°C. According to the different dilution times, the above procedures were repeated until the plate counts reached 100–300 single colonies. The number of colonies was counted and multiplied by the dilution.

### Grouping and Treatment of Animals

After 3 days of adaptive feeding, the mice were randomly assigned to five groups (*n* = 6), including the blank, negative control, atorvastatin, DSM20016, and HI120 groups. In the blank group, mice were fed a normal diet for 12 weeks. negative-control group mice were fed a high-fat diet for 12 weeks. After 4 weeks, sunflower seed oil (containing 60% LA) (20 μL) was administered by gavage daily (every 2 days after 8 weeks). Mice in the atorvastatin group were fed a high-fat diet for 12 weeks; after 4 weeks, sunflower seed oil (20 μL) + atorvastatin (0.18 mg/kg) was administered by gavage daily (every 2 days after 8 weeks). In the DSM20016 group, the mice were fed a high-fat diet for 12 weeks; after 4 weeks, sunflower seed oil (20 μL) + DSM20016 bacterial powder (1,000 mg/kg) was administered by gavage daily (every 2 days after 8 weeks). HI120 group mice were fed a high-fat diet for 12 weeks. After 4 weeks, sunflower seed oil (20 μL) + HI120 bacterial powder (1,000 mg/kg) was administered by gavage daily (every 2 days after 8 weeks). Because atorvastatin is most effective when administered at night, the last four groups of mice were treated at 20:00 h every night.

### Grouping and Treatment of Cells

#### Cholesterol Synthesis-, Absorption-, Excretion-, and Expression-Related Genes

Control group HepG2/Caco-2 cells were not treated. In the LA group, HepG2/Caco-2 cells were incubated with LA (0.7 μL/mL) for 48 h. In the DSM20016 group, LA (0.7 μL/mL) was incubated with DSM20016 solution (number of viable bacteria was approximately 1.0 × 10^7^ cfu/mL) for 48 h. After centrifugation at 5,000 rpm, the supernatant was extracted (ensuring that the initial concentration of LA in the solution was 0.7 μL/mL) and incubated with HepG2/Caco-2 cells for 48 h. In the HI120 group, LA (0.7 μL/mL) was incubated with HI120 solution (number of viable bacteria was ~1.0 × 10^7^ cfu/mL) for 48 h. After centrifugation at 5,000 rpm, the supernatant was extracted (initial concentration of LA in the solution is 0.7 μL/mL) and then incubated with HepG2/Caco-2 cells for 48 h.

#### NPC1L1/HMG-CR Expression Following siRNA Interference Targeting SREBP-2

In the NC group, HepG2/Caco-2 cells were transfected with negative-control siRNA. In siRNA group 1, HepG2/Caco-2 cells were transfected with the siRNA of SREBP-2 gene fragment 1. In siRNA group 2, HepG2/Caco-2 cells were transfected with the siRNA of SREBP-2 gene fragment 2. SREBP-2 siRNA primer sequence: 5′-AGCAGCAGCAGCAGCAATGG-3′ (forward), 5′-GCCGCCGAGGGAGAGAAGG-3′ (reverse).

Each transfected sample was prepared as follows: (1) siRNA dilution: 1.25 μL 20 μM siRNA stock solution was diluted with 30 μL Ribofect TM CP buffer (RiboBio Co., Ltd., Wuhan, China) and mixed gently. (2) Mixed solution: 3 μL Ribofect TM CP reagent (RiboBio Co., Ltd.) was added. The solution was gently blown and mixed, incubated at room temperature for 0–15 min, and used to prepare the transfection complex. (3). The Ribofect TM CP transfection complex was added to a suitable amount of complete culture medium without double antibody and mixed gently. Transfection step: cells were inoculated and spread onto a 6-well plate at a density of 5 × 10^5^ cells per well and transfected at ~70% confluence. The final concentration of siRNA was 50 nM. Subsequently, 1,863 μL medium, 120 μL buffer, 5 μL siRNA, and 12 μL Regent were added to each pore and then mixed. The cells were cultured in an incubator for 48 h. The efficiency of siRNA silencing after transfection was assessed.

#### Immunofluorescence Analysis of Cholesterol Absorption and NPC1L1/SREBP-2 Expression

In the NC group, Caco-2 cells were incubated with Cholesteryl Bodipy^TM^ FL C12 (20 μM/L) for 48 h. In the LA group, Caco-2 cells were incubated with Cholesteryl Bodipy^TM^ FL C12 (20 μM/L) and LA (0.7 μL/mL) for 48 h. In the DSM20016 group, LA (0.7 μL/mL) was incubated with DSM20016 solution (number of viable bacteria was ~1.0 × 10^7^ cfu/mL) for 48 h. After centrifugation at 5,000 rpm, the supernatant was extracted (ensuring that the initial concentration of LA in the solution was 0.7 μL/mL). Caco-2 cells were then incubated with Cholesteryl Bodipy^TM^ FL C12 (20 μM/L) for 48 h. In the HI120 group, LA (0.7 μL/mL) was added to HI120 solution (number of viable bacteria was ~1.0 × 10^7^ cfu/mL) and incubated for 48 h, After centrifugation at 5,000 rpm, the supernatant was extracted (ensuring that the initial concentration of LA in the solution was 0.7 μL/mL). Caco-2 cells were then incubated with Cholesteryl Bodipy^TM^ FL C12 (20 μM/L) for 48 h.

#### Detection of Cholesterol Absorption by Immunofluorescence and NPC1L1 Expression After siRNA Silencing of SREBP-2

In the NC group, Caco-2 cells were incubated with Cholesteryl Bodipy^TM^ FL C12 (20 μM/L) for 48 h. In the SREBP-2 RNAi 1 group, Caco-2 cells were transfected with the siRNA of SREBP-2 gene fragment 1 and incubated with Cholesteryl Bodipy^TM^ FL C12 (20 μM/L) for 48 h. In the SREBP-2 RNAi 2 group, Caco-2 cells were transfected with the siRNA of SREBP-2 gene fragment 2 and incubated with Cholesteryl Bodipy^TM^ FL C12 (20 μM/L) for 48 h. The transfection procedure was performed as described above.

### Detection Index of Animal- and Cell-Based Experiments

After 28 days, the body weight, food intake, and water intake of the mice were measured every 7 days. At the end of the experiment, the mice were anesthetized by intraperitoneal injection of sodium pentobarbital (1%, 40 mg/kg), blood was collected from the heart, and the mice were sacrificed. Tissue samples including small intestinal and liver tissues were collected from the same segment or lobe of each mouse and stored at −80°C. The blood was incubated at room temperature for 1 h and centrifuged at 3,000 rpm for 15 min, after which the serum was separated. The serum total cholesterol and triglyceride levels (HITACHI 7150 automatic biochemical analyzer, Tokyo, Japan) were then measured.

Total RNA was extracted from the liver/small-intestinal tissue of animals as well as from the cell lines. In both sample types, extraction was performed by the TRIzol method. The expression of NPC1L1, SREBP-2, HMG-CR, CYP7A1, LXR, and FXR was measured by RT-qPCR using the following primers: 51v 5′-TGACAAGTTCCAGGTTGCGT-3′ (forward), 5′-TAGCTGACGGCAAAGACAGG-3′ (reverse); 5′-ATGGACGACAGCGGCGAGC-3′ (forward), 5′-CAGAAGAATCCGTGAGCGGTCTAC-3′ (reverse); 5′-GATCCAGGAGCGAACCAAGAGAG-3′ (forward), 5′-GCTACAGAAGCCCCAAGCACAA-3′ (reverse); 5′-CAAGACCGCACATAAAGCC-3′ (forward), 5′-GATGCCCAGAGGATCACG-3′ (reverse); 5′-GATGCCCAGAGGATCACG-3′ (forward), 5′-GCTGACTCCAACCCTATCCC-3′ (reverse) and 5′-GTGAGGGCTGCAAAGGTTTC-3′ (forward), 5′-CAAACCTGTATACATACATTCAGCC-3′ (reverse). Their mRNA levels were presented as the ratio of the expression of each gene to GAPDH, in which its primers were 5′-GGACCTCATGGCCTACATGG-3′ (forward) and 5′-TAGGGCCTCTCTTGCTCAGT-3′ (reverse).

Total protein was extracted both from the liver/small intestine tissue of animals and from cell lines. The protein levels of NPC1L1, SREBP-2, HMG-CR, CYP7A1, LXR, and FXR were measured by western blotting using the following antibodies: anti-NPC1L1(Cat: PA1-16800, Thermo Fisher Scientific, Waltham, MA, USA), anti-SREBP-2 (Cat: ab28482, Abcam, Cambridge, UK), anti-HMG-CR (Cat: 3952-100, Bio Vision, San Francisco, CA, USA), anti-CYP7A1 (Cat: sc14423, Santa Cruz Biotechnology, Dallas TX, USA), LXR (Cat: GTX89656, GeneTex, Irvine, CA, USA), FXR (Cat: GTX113867, GeneTex, CA, USA), anti-GAPDH (Cat: AC027, ABclonal Technology, Wuhan, China), anti-rabbit secondary antibody (Cat: orb229657, Biorbyt, Cambridge, UK), anti-goat secondary antibody (Cat: 023002, Kangwei, Beijing, China).

Cells in the middle pores of the confocal dish were fixed, permeabilized, sealed, incubated with primary and secondary antibodies, stained with DAPI, and observed under a confocal laser scanning microscope (28).

### Statistical Analysis

All cell experiments were performed three times independently and each experiment was performed in triplicate. GraphPad Prism 7 software (GraphPad, Inc., La Jolla, CA, USA) was used to prepare various statistical charts. The results obtained were expressed as the mean ± SD. Statistical analysis was performed by one-way analysis of variance followed by Tukey's multiple comparisons test for multiple comparison. *P* < 0.05 was considered as statistically significant.

## Results

### Morphological Characteristics of *L. reuteri* HI120

**Figure 1:**
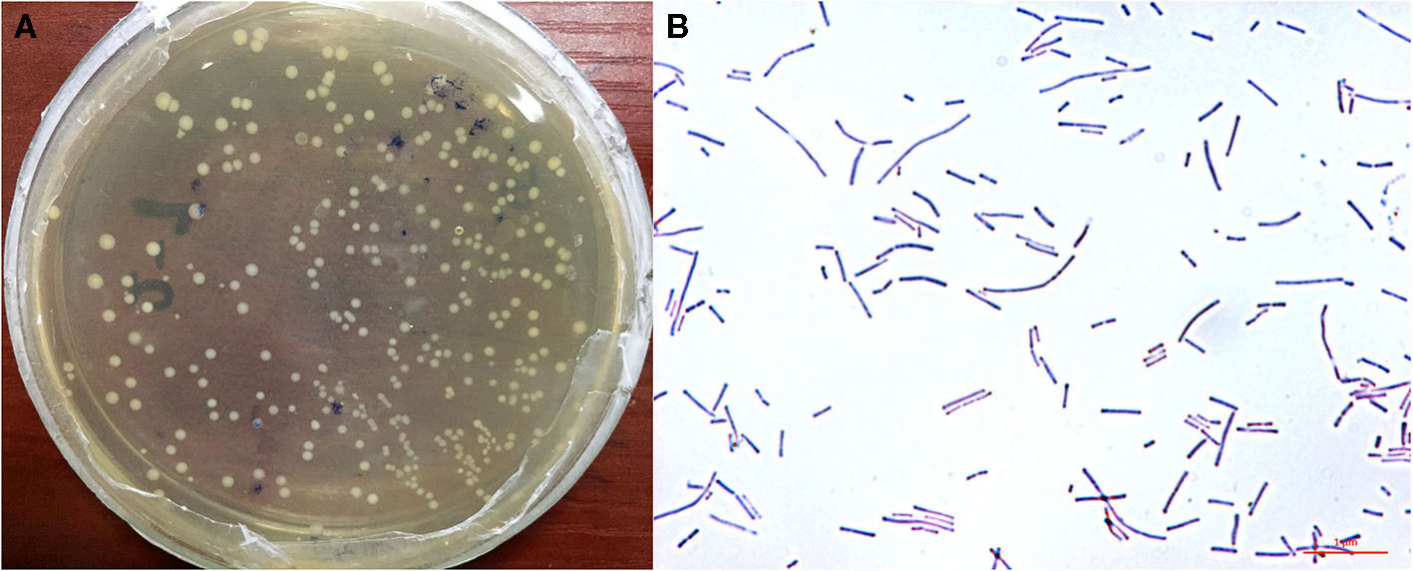
Morphological characteristics and observations of *Lactobacillus reuteri* HI120 under an optical microscope. **(A)** Morphological characteristics. **(B)** Observations after Gram staining (1,000× magnification).

Analysis of the morphological characteristics of HI120 under an optical microscope (Figures 1A,B) revealed that HI120 is a slightly irregular type of campylobacter with a rounded end.

### Sequencing Results of *L. reuteri* HI120

**Figure 2:**
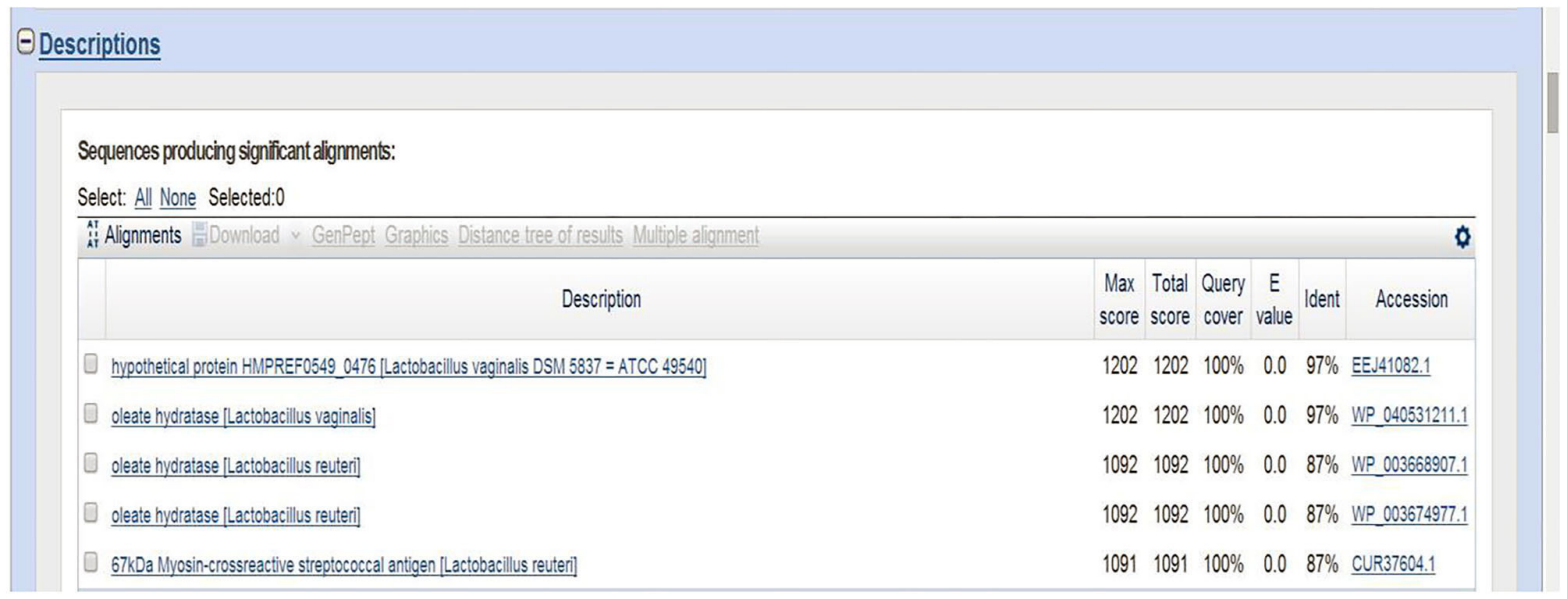
Comparison of LAI amino acid sequences of HI120 and other *Lactobacillus reuteri* strains.

The 16S rRNA sequence of HI120 was 99% (Table 1) homologous with those of other *L. reuteri* strains. Thus, HI120 belongs to *L. reuteri*, which is supported by its biological characteristics. The LAI amino acid sequence of HI120 was 87% homologous with that of a standard strain of *L. reuteri* (DSM20016) and 97% homologous with that of *Lactobacillus vaginalis* (Figure 2). Therefore, HI120 may express high levels of LAI.

**Table 1:** Comparison of 16S rRNA sequences of HI120 and other strains.

**Description**	**Max score**	**Total score**	**Query cover**	**E value**	**Ident**	**Accession**
*Lactobacillus reuteri* DSM 20016, complete genome	2,804	16,737	100%	0.0	99%	CP000705.1
*Lactobacillus reuteri* strain I49, complete genome	2,798	16,742	100%	0.0	99%	CP015408.1
*Lactobacillus reuteri* TD1, complete genome	2,793	16,720	100%	0.0	99%	CP006603.1
*Lactobacillus reuteri* I5007, complete genome	2,793	16,709	100%	0.0	99%	CP006011.1
*Lactobacillus reuteri* strain IRT, complete genome	2,804	16,731	100%	0.0	99%	CP011024.1

### LA–CLA Conversion Rate of *L. reuteri* HI120

**Figure 3:**
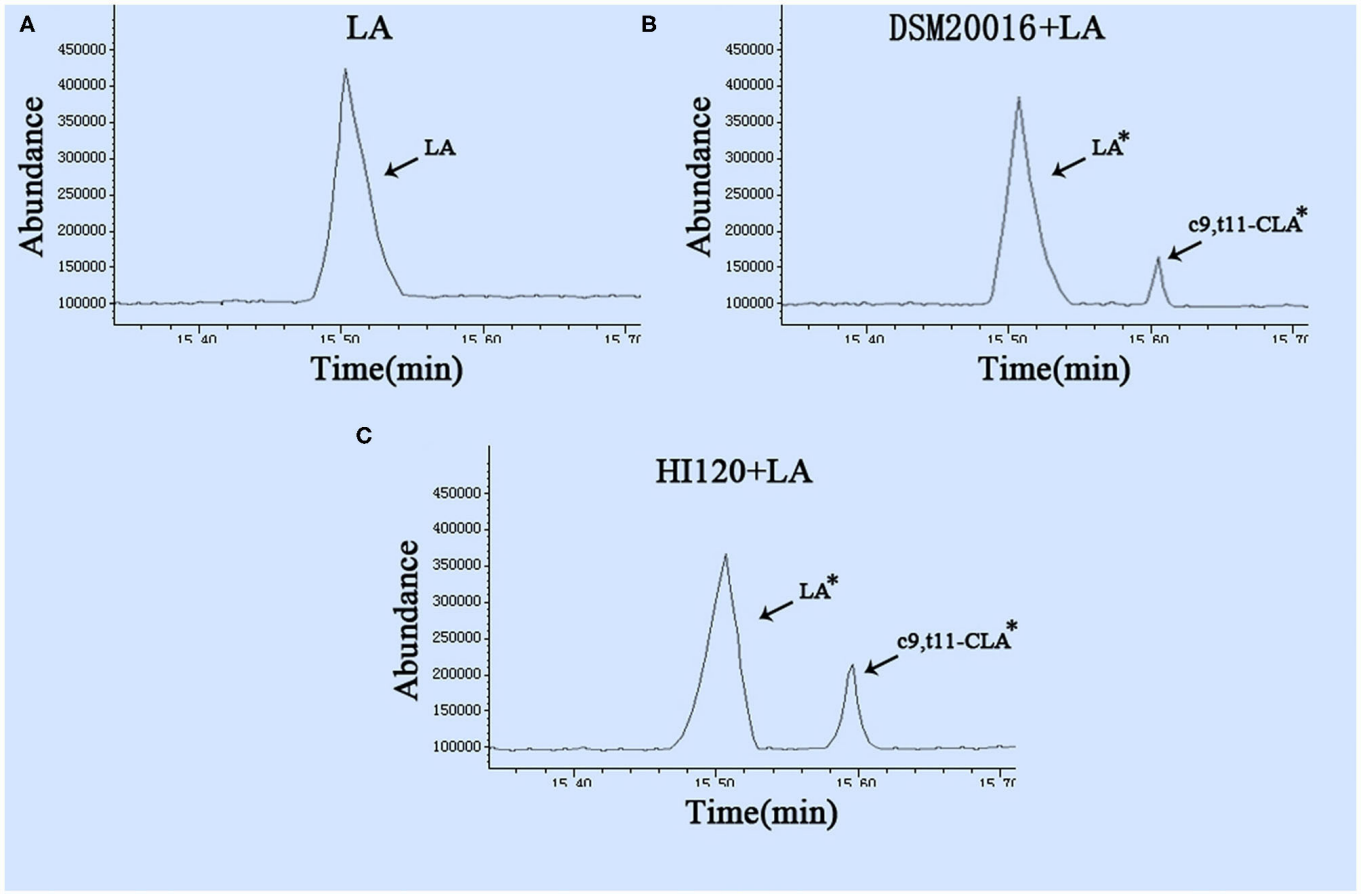
Gas chromatogram in all groups **(A)** LA group. **(B)** DSM20016 + LA group. **(C)** HI120 group + LA. *p < 0.05 compared to the LA group.

**Figure 4:**
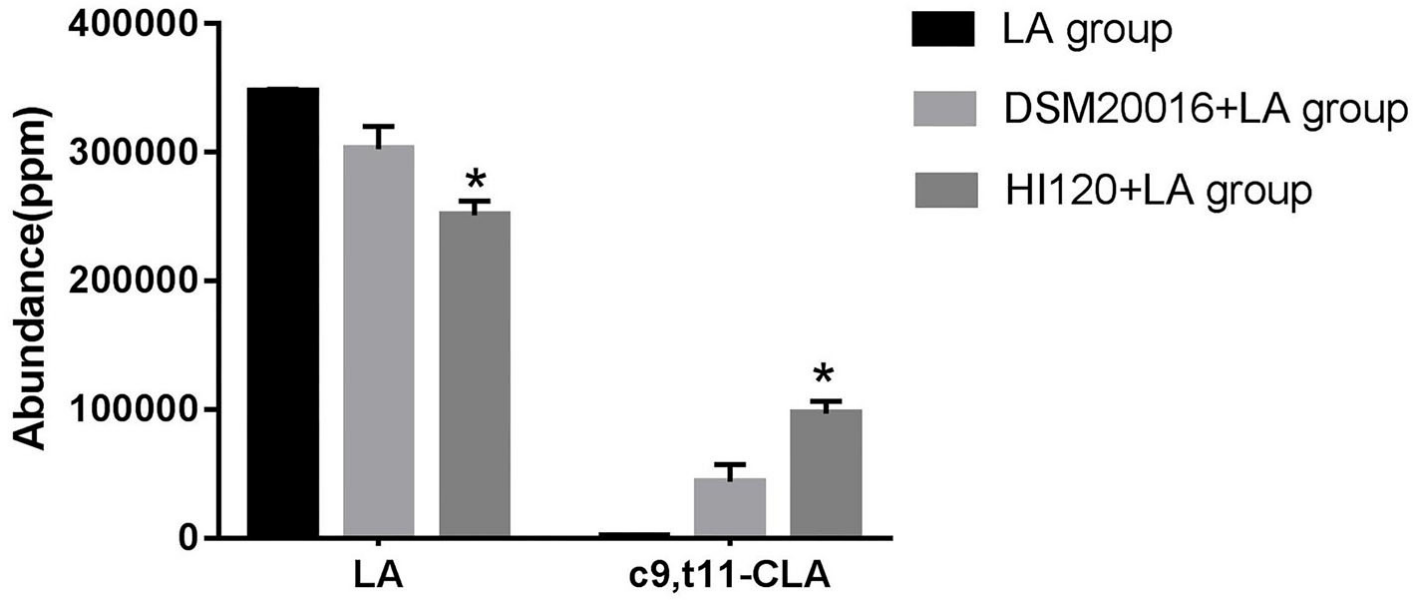
Abundance levels in the gas chromatogram of all groups. LA group. DSM20016 + LA group. HI120 + LA group. *p < 0.05 compared to the DSM20016+LA group.

Gas chromatography (29) revealed that the main chemical structure of CLA generated by HI120 and DSM20016 was c9, t11-CLA (Figure 3). The conversion rate of LA to CLA was 27.63% for HI120 and 12.54% for DSM20016. The former rate was ~2.2-fold higher (Figure 4) than that of DSM20016.

### Food Intake, Water Intake, Body Weight, and Total Cholesterol and Triglyceride Serum Levels of Animals

**Figure 5:**
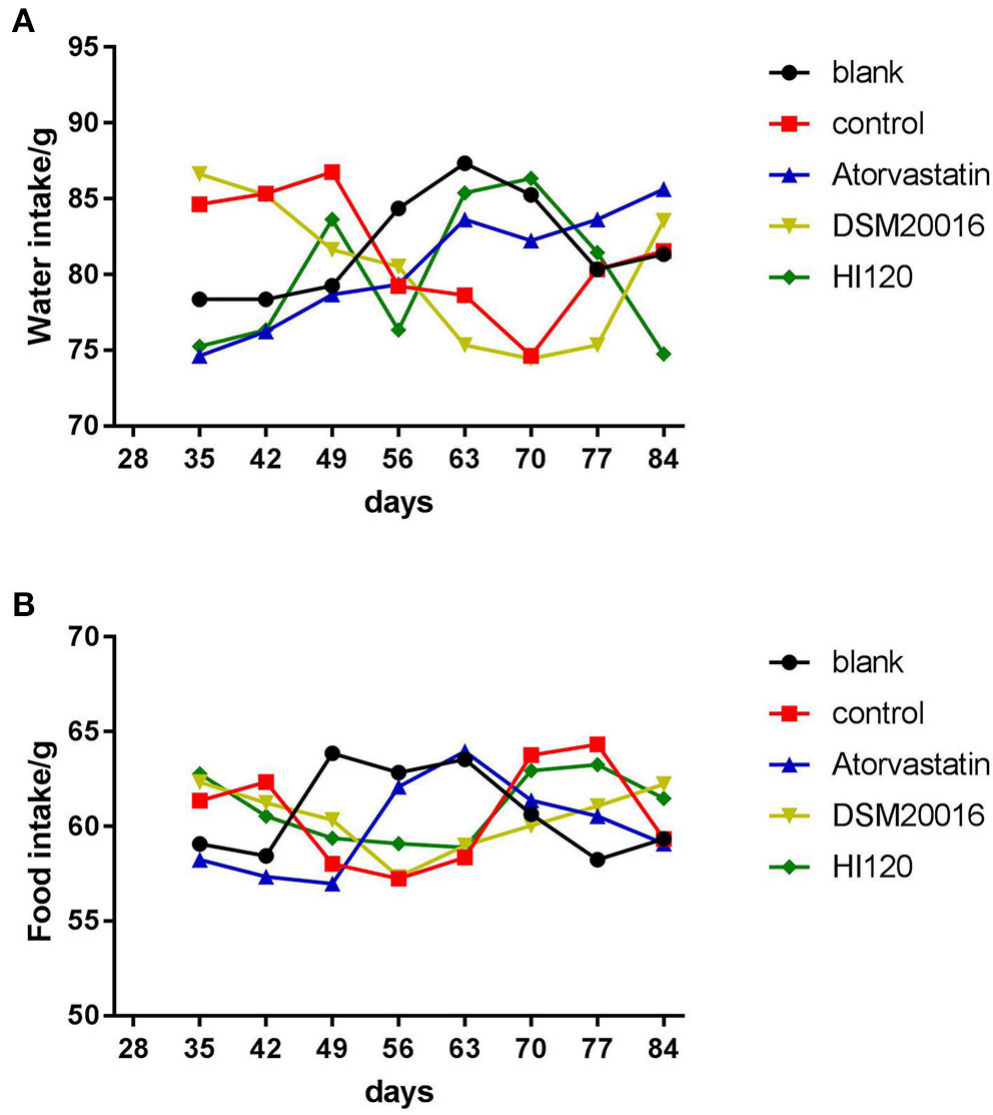
Water and food intake in all groups. **(A)** Water intake. **(B)** Food intake.

**Figure 6:**
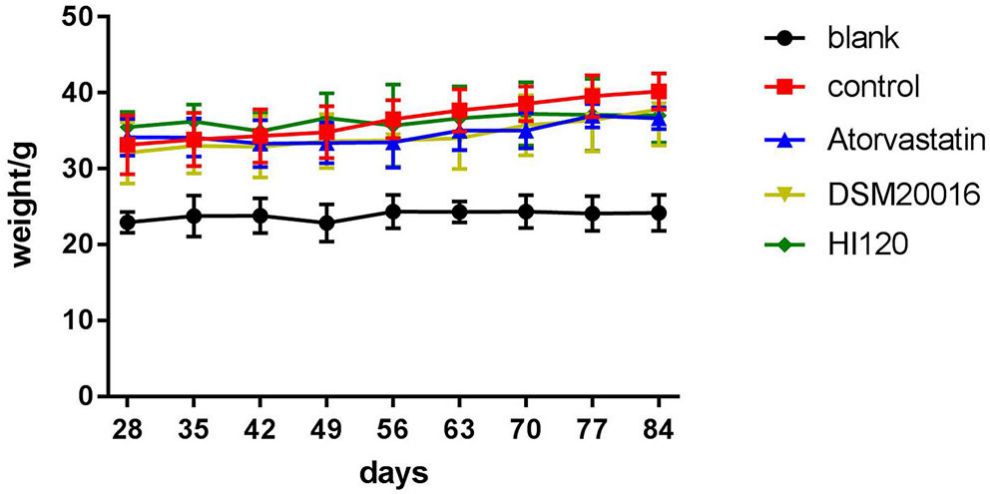
Body weight changes in all groups.

**Figure 7:**
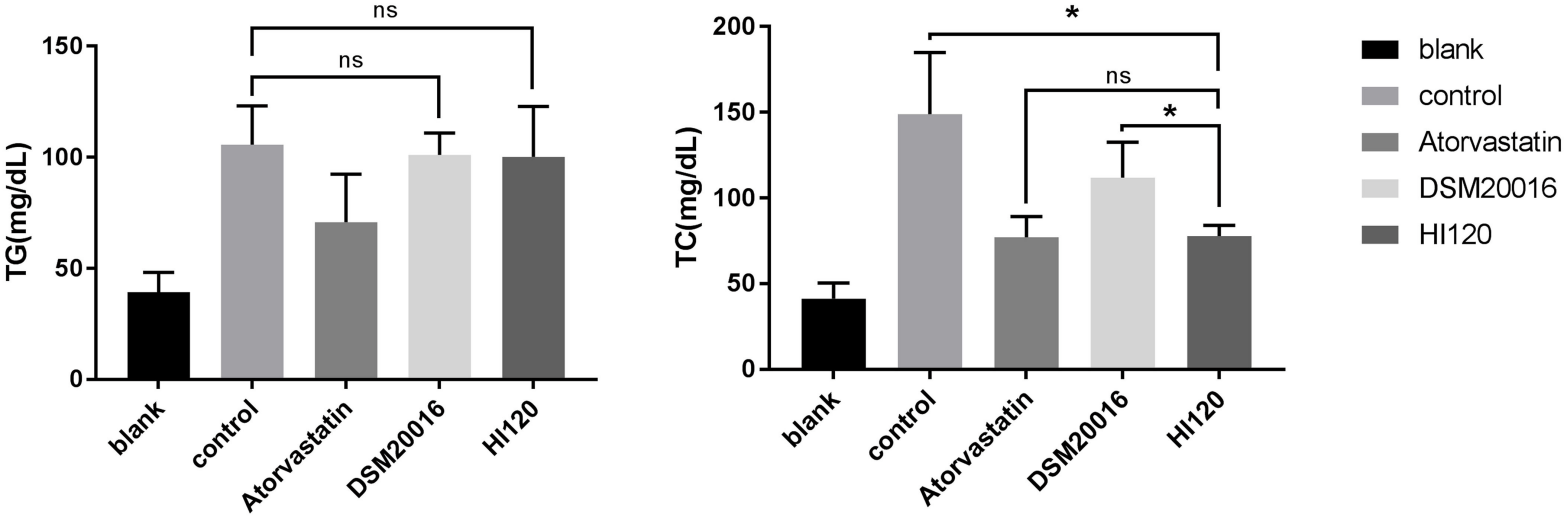
Serum triglyceride and total cholesterol levels in in all groups.*p < 0.05.

There was no significant difference in the diet and water intake between experimental groups (Figures 5A,B). Additionally, no significant difference in body weight was observed between mice in the four experimental groups on a high-fat diet and body weight of mice on a regular diet (Figure 6, Table 2). The triglyceride levels of mice fed a high-fat diet were all increased; however, the levels in the mice fed with atorvastatin were decreased, whereas those fed HI120 and DSM20016 displayed no significant change. The cholesterol level of mice fed HI120 decreased as much as that of the level of the atorvastatin group mice and was significantly lower than that of DSM20016 group mice (Figure 7, Table 3). Mice fed HI120 did not lose weight or showed changes in triglycerides; however, cholesterol levels were decreased.

**Table 2:** Body-weight changes in all groups (mean ± SD, *n* = 6).

	**Weight (g)**				
**Time**	**Blank**	**Control**	**Atorvastatin**	**DSM20016**	**HI120**
28 day	22.932 ± 1.389	33.143 ± 3.876^*^	34.127 ± 2.425^*^	32.093 ± 4.034^*^	35.467 ± 2.000^*^
35 day	23.783 ± 2.708	33.850 ± 3.498^*^	34.087 ± 2.503^*^	33.018 ± 4.034^*^	36.218 ± 2.203^*^
42 day	23.820 ± 2.295	34.295 ± 3.500^*^	33.323 ± 3.099^*^	32.893 ± 4.051^*^	34.940 ± 2.389^*^
49 day	22.855 ± 2.441	34.803 ± 3.412^*^	33.412 ± 2.680^*^	33.630 ± 3.559^*^	36.692 ± 3.274^*^
56 day	24.355 ± 2.225	36.508 ± 2.497^*^	33.464 ± 3.345^*^	33.743 ± 3.569^*^	35.703 ± 5.431^*^
63 day	24.320 ± 1.366	37.698 ± 2.818^*^	35.008 ± 2.558^*^	34.040 ± 4.042^*^	37.215 ± 4.178^*^
70 day	24.347 ± 2.192	38.575 ± 2.305^*^	35.000 ± 2.349^*^	35.733 ± 3.972^*^	37.216 ± 4.178^*^
77 day	24.105 ± 2.300	39.573 ± 2.760^*^	36.960 ± 1.552^*^	36.378 ± 4.131^*^	37.117 ± 4.757^*^
84 day	24.187 ± 2.355	40.183 ± 2.400^*^	36.645 ± 1.437^*^	37.797 ± 4.765^*^	37.040 ± 3.601^*^

* *p < 0.05 compared with the blank group*.

**Table 3:** Serum TG and TC levels in in all groups (mmol/L) (mean ± SD, *n* = 6).

	**Blank**	**Control**	**Atorvastatin**	**DSM20016**	**HI120**
TG	39.333 ± 8.802	105.667 ± 17.512	70.833 ± 21.591^*^	101.000 ± 9.960	100.167 ± 22.825
TC	41.167 ± 9.283	148.833 ± 36.041	77.000 ± 12.313^*^	111.833 ± 20.760^*^	77.667 ± 6.377^**^

* *p < 0.05 compared with the control group*.

** *p < 0.05 compared with the DSM20016 group*.

### Expression Analyses (Real-Time PCR and Western Blotting) in Animals

**Figure 8:**
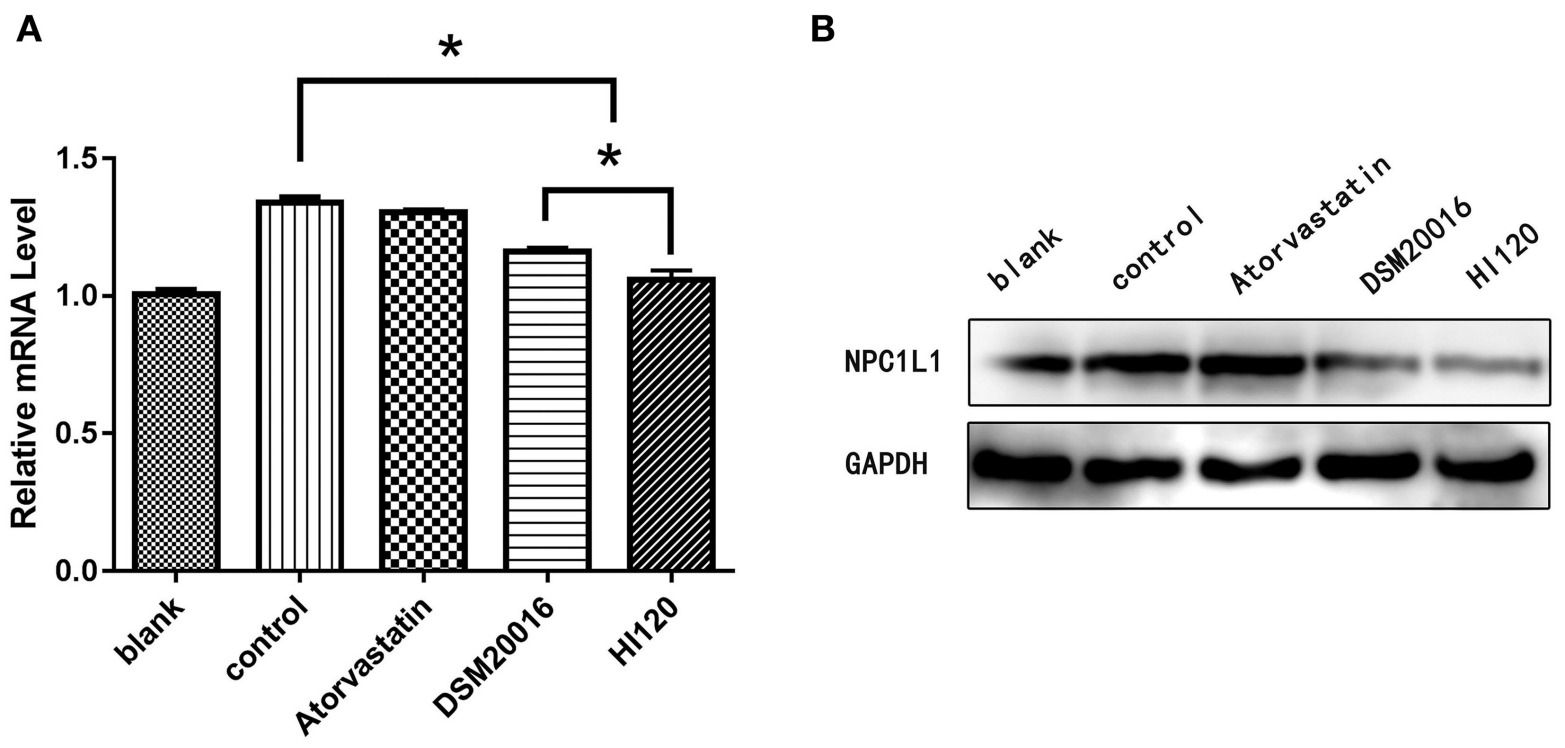
mRNA and protein expression levels of *NPC1L1* in all groups. **(A)** qRT-PCR results. **(B)** Western blotting results. *p < 0.05.

**Figure 9:**
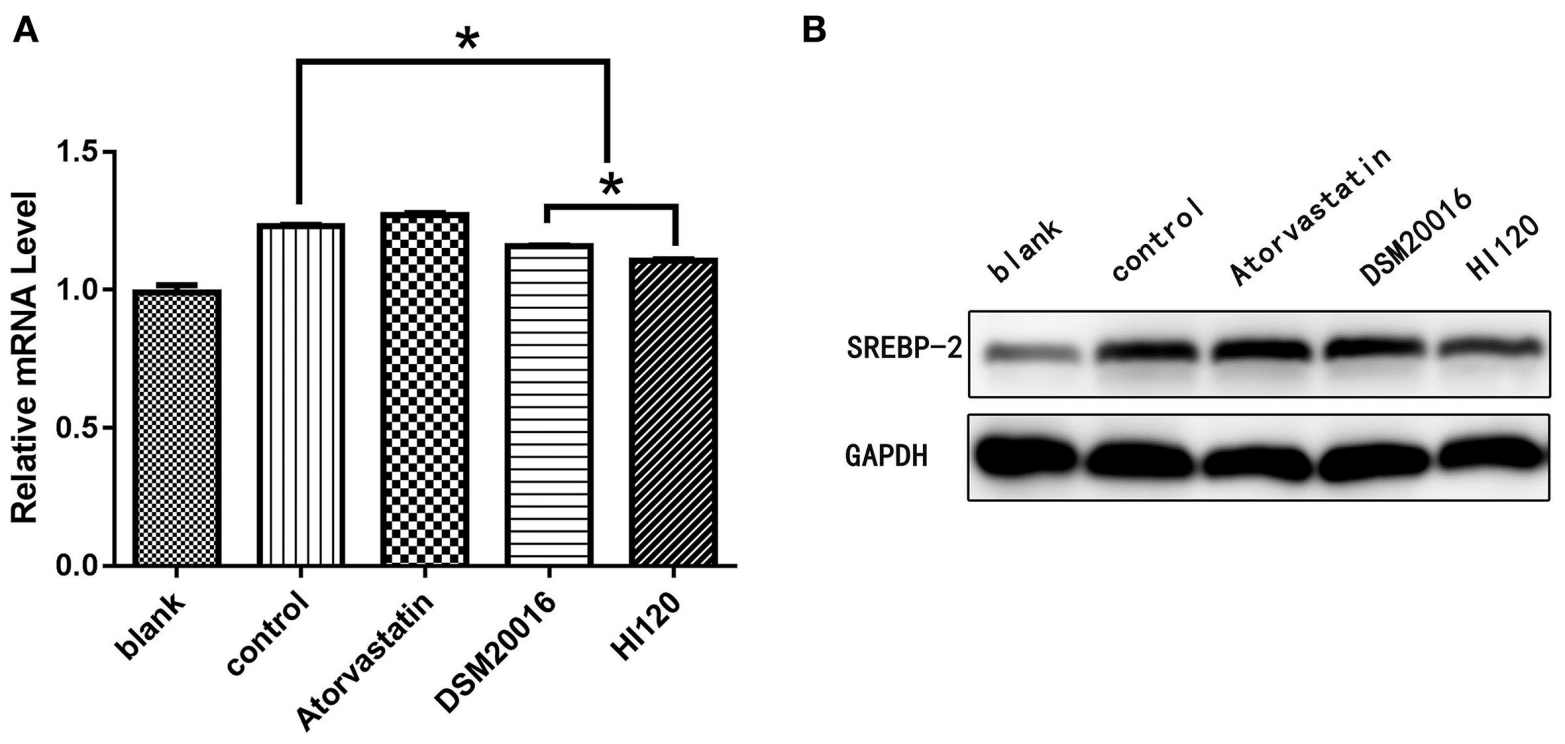
mRNA and protein expression levels of *SREBP-2* in all groups. **(A)** qRT-PCR results. **(B)** Western blotting results. *p < 0.05.

**Figure 10:**
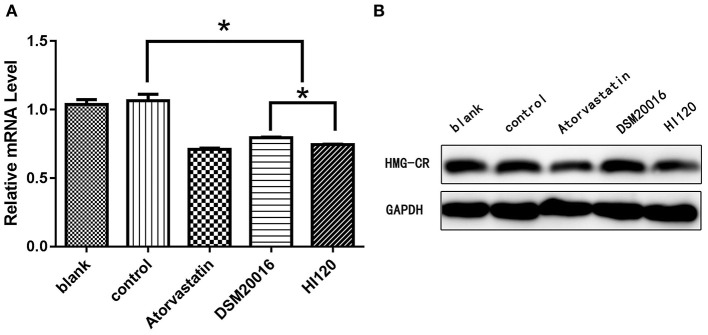
mRNA and protein expression levels of *HMG-CR* in all groups. **(A)** qRT-PCR results. **(B)** Western blotting results. *p < 0.05.

**Figure 11:**
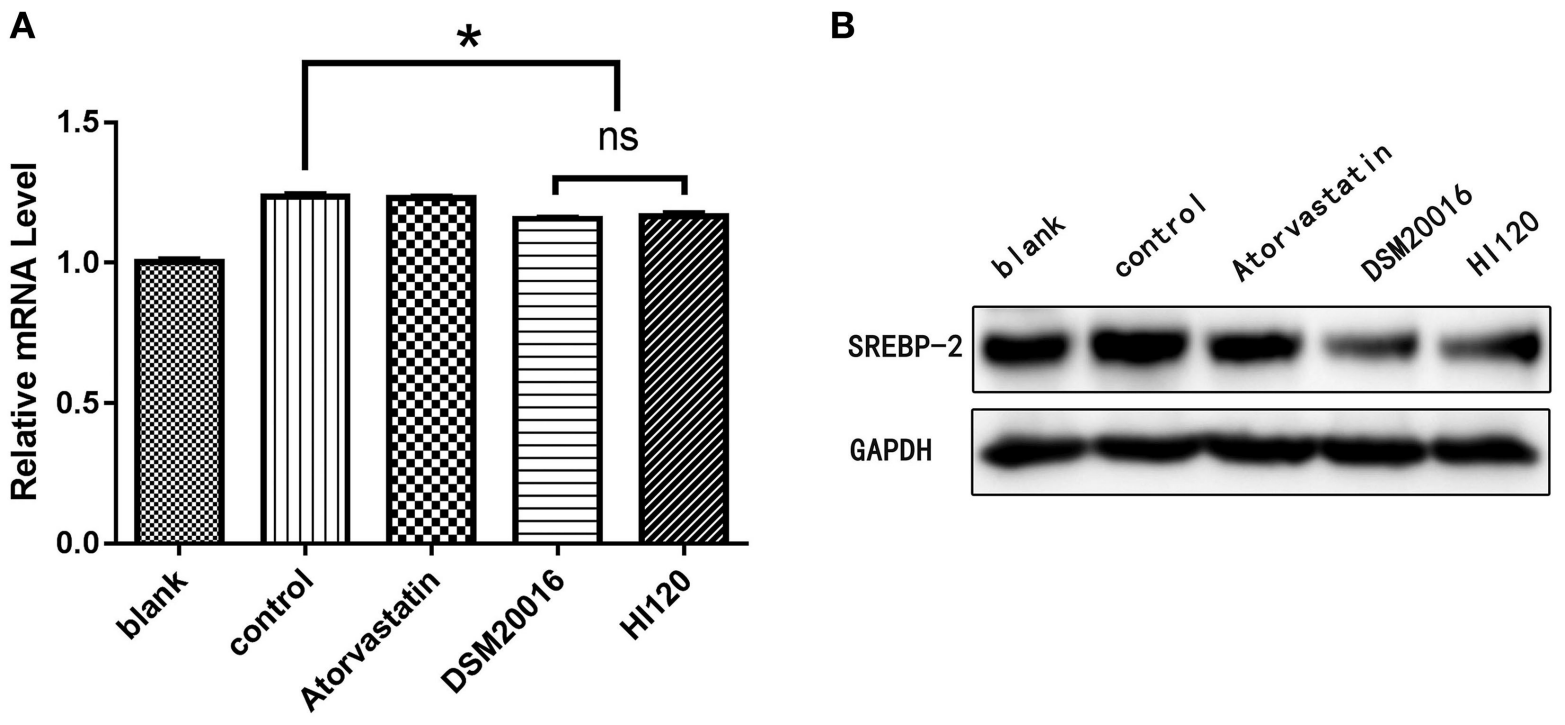
mRNA and protein expression levels of *SREBP-2* in all groups. **(A)** qRT-PCR results. **(B)** Western blotting results. *p < 0.05.

**Figure 12:**
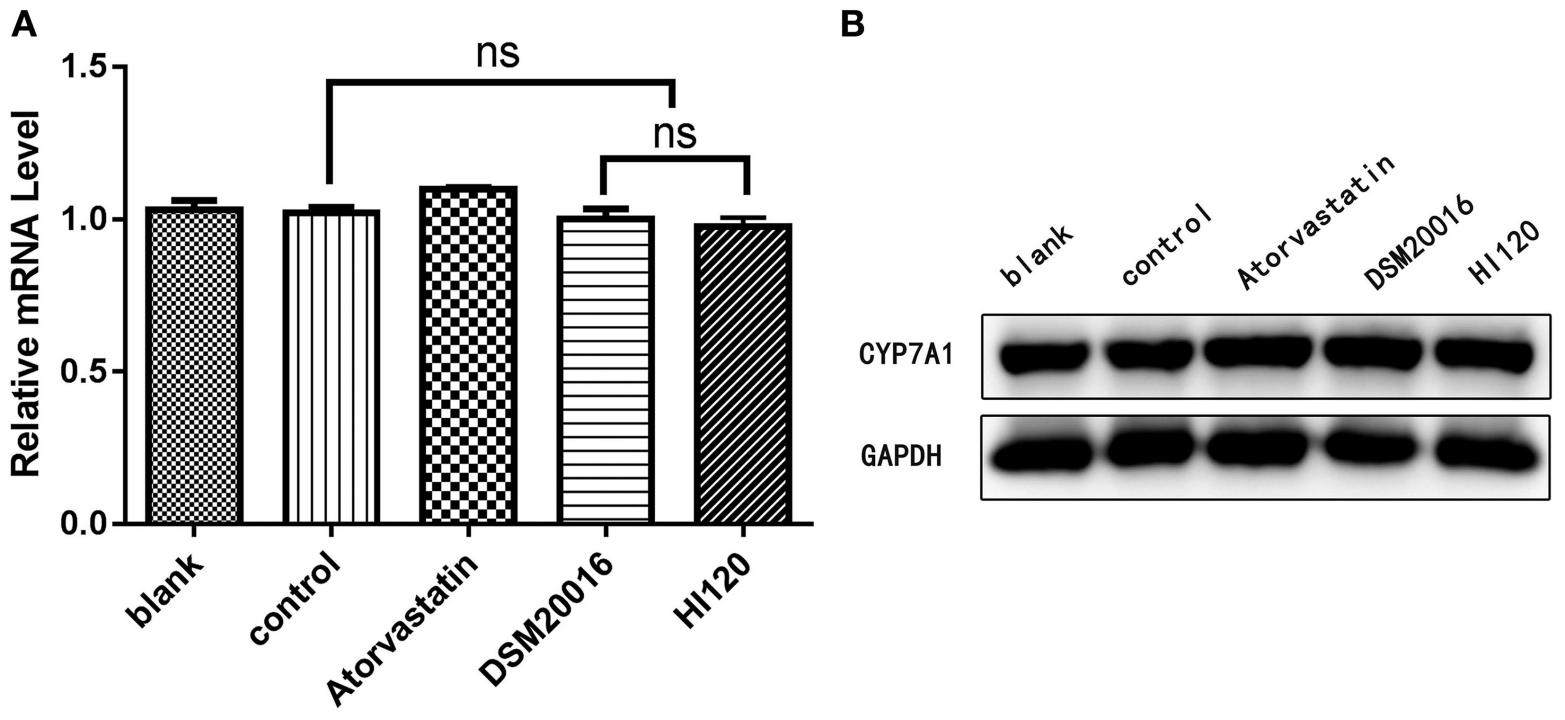
mRNA and protein expression levels of *CYP7A1* in all groups. **(A)** qRT-PCR results. **(B)** Western blotting results. *p < 0.05.

**Figure 13:**
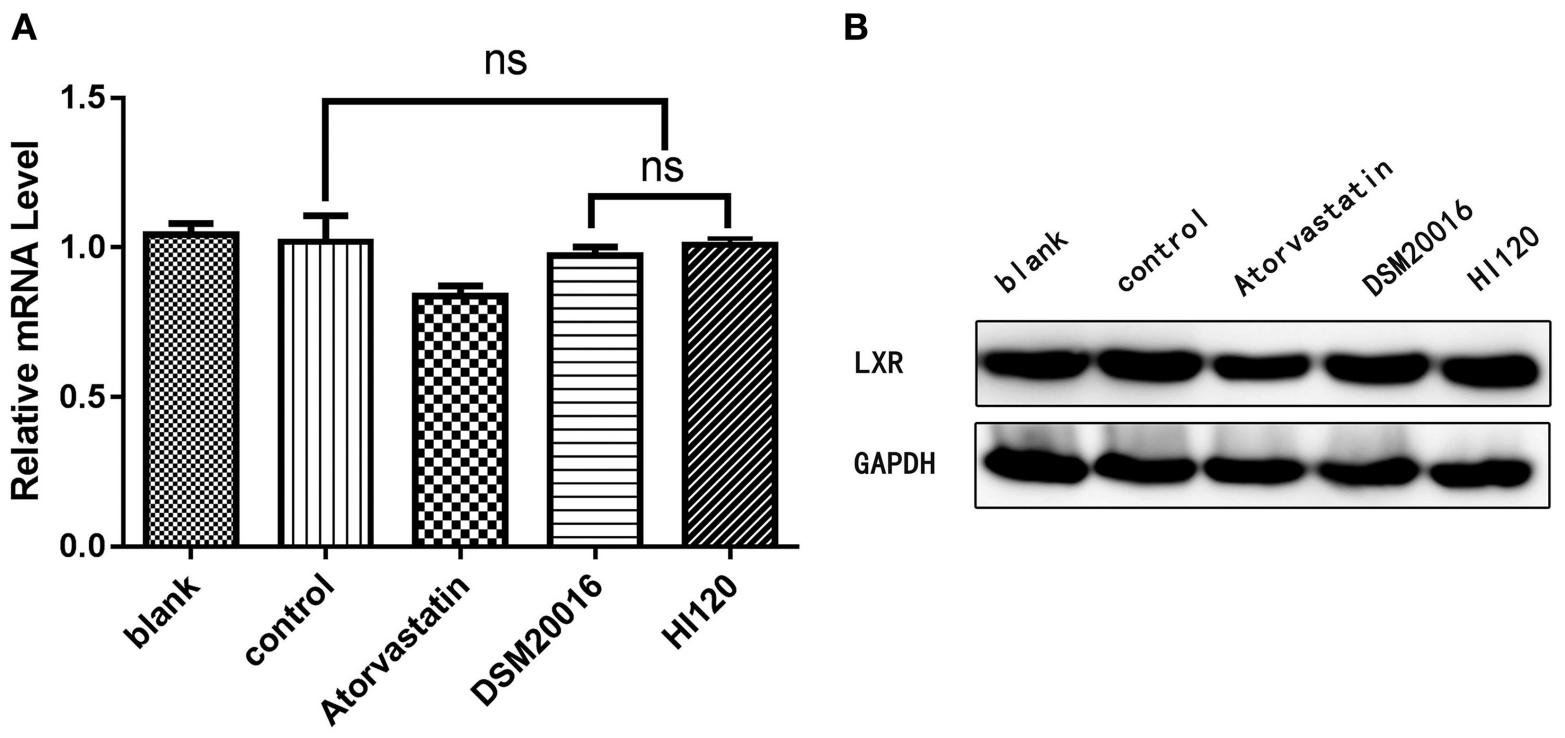
mRNA and protein expression levels of *LXR* in all groups. **(A)** qRT-PCR results. **(B)** Western blotting results. *p < 0.05.

**Figure 14:**
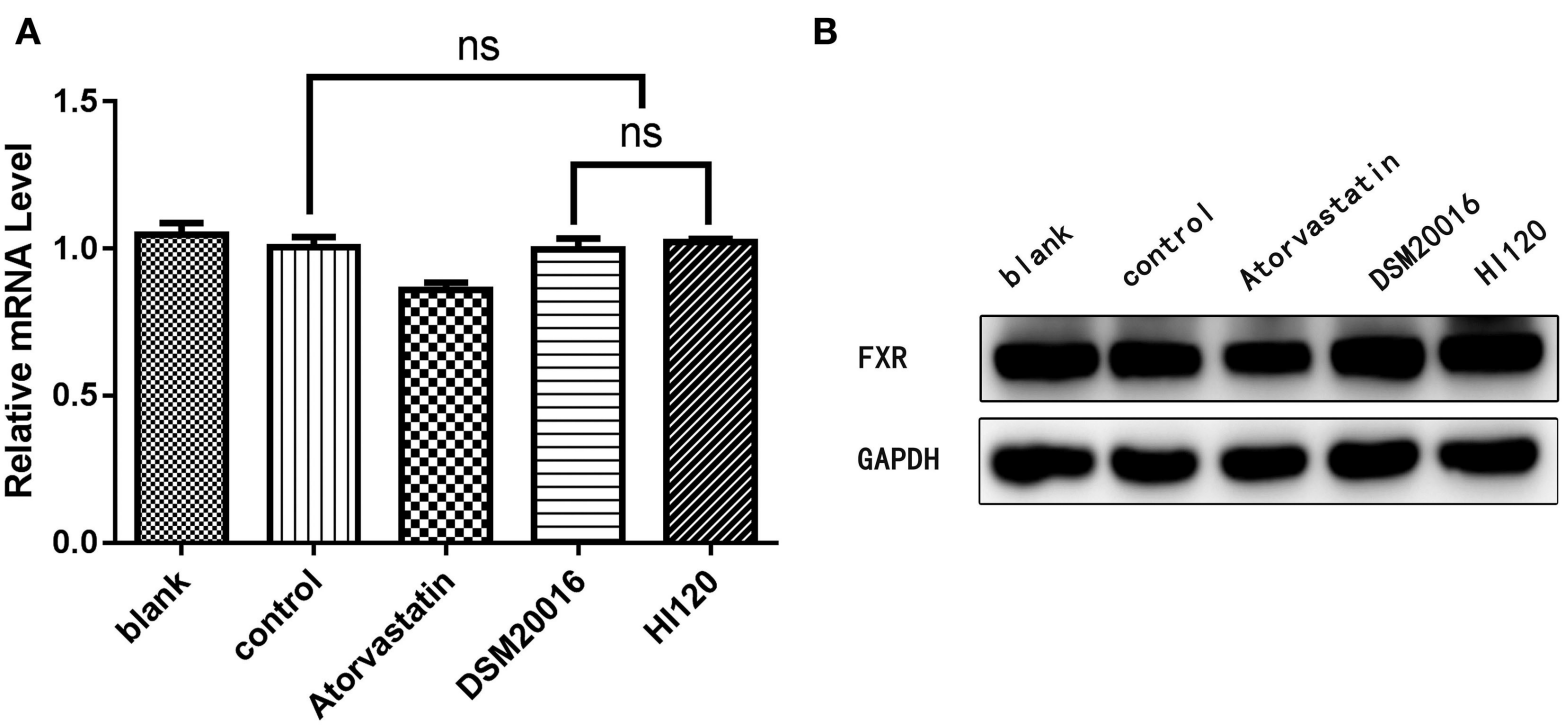
mRNA and protein expression levels of *FXR* in all groups. **(A)** qRT-PCR results. **(B)** Western blotting results. *p < 0.05.

Compared with the control group parameters, the mRNA levels and protein expression of *NPC1L1* and *SREBP-2* were significantly decreased in the small intestine of mice in the HI120 and DSM20016 groups; however, these levels in the HI120 group were lower than those in the DSM20016 group (Figures 8, 9). Compared with control group parameters, the mRNA and protein expression levels of *HMG-CR* in the liver of mice in the HI120 and DSM20016 groups were significantly lower; however, HI120 group levels were lower than those in the DSM20016 group (Figure 10). Compared with control group parameters, the mRNA and protein expression levels of *SREBP-2* in the liver of mice in the HI120 and DSM20016 groups were significantly lower, but there was no significant difference between the HI120 and DSM20016 groups (Figure 11). Compared with control group parameters, the mRNA and protein expression levels of *CYP7A1, LXR*, and *FXR* in the liver of mice in the HI120 and DSM20016 groups were not significantly different; there was also no significant difference observed between the HI120 and DSM20016 groups (Figures 12–14).

### Expression Analyses (Real-Time PCR and Western Blotting) of Cells

#### Expression of Genes Related to Cholesterol Synthesis, Absorption, and Metabolism

**Figure 15:**
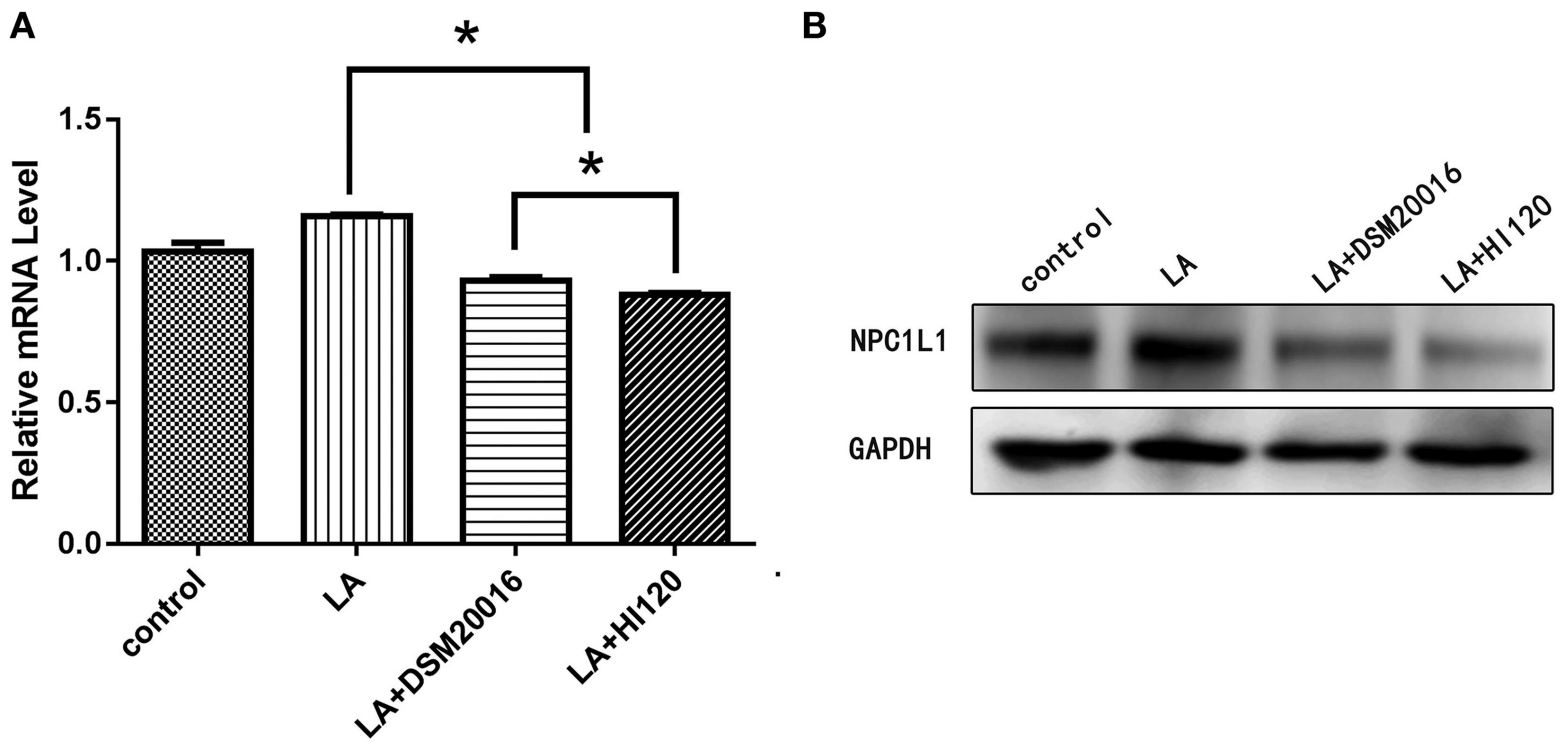
mRNA and protein expression levels of *NPC1L1* in all groups. **(A)** qRT-PCR results. **(B)** Western blotting results. *p < 0.05.

**Figure 16:**
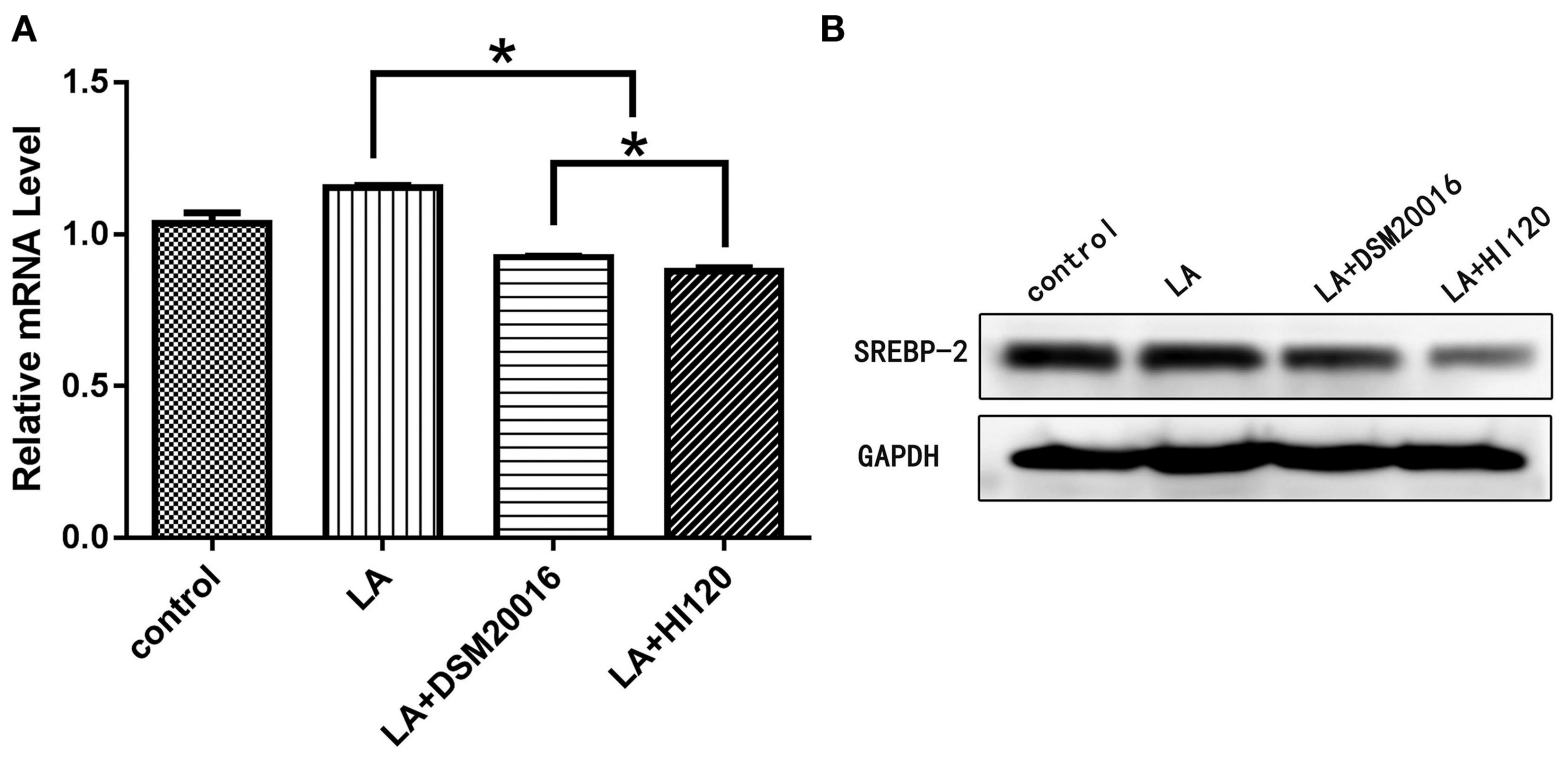
mRNA and protein expression levels of *SREBP-2* in all groups. **(A)** qRT-PCR results. **(B)** Western blotting results. *p < 0.05.

**Figure 17:**
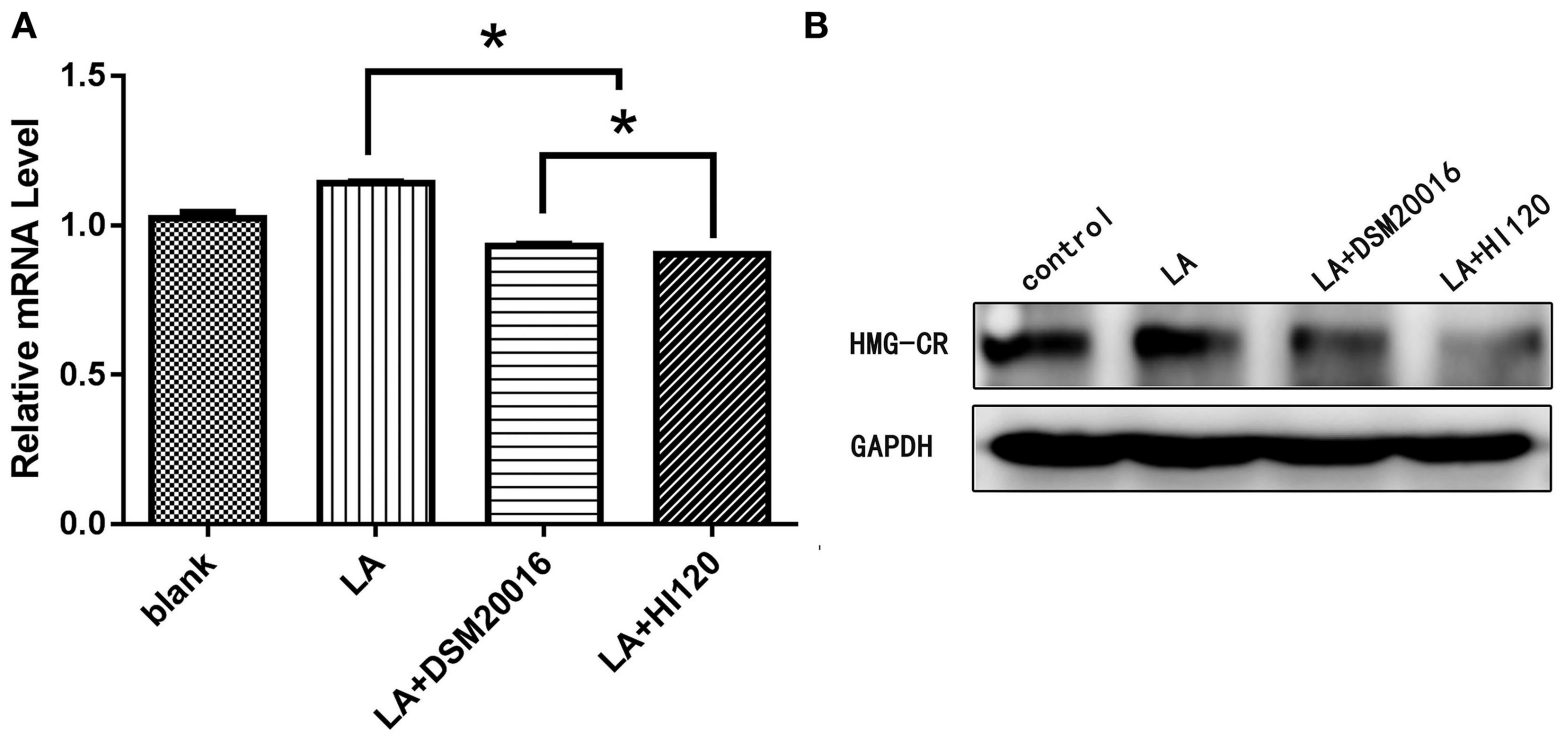
mRNA and protein expression levels of *HMG-CR* in all groups. **(A)** qRT-PCR results. **(B)** Western blotting results. *p < 0.05.

**Figure 18:**
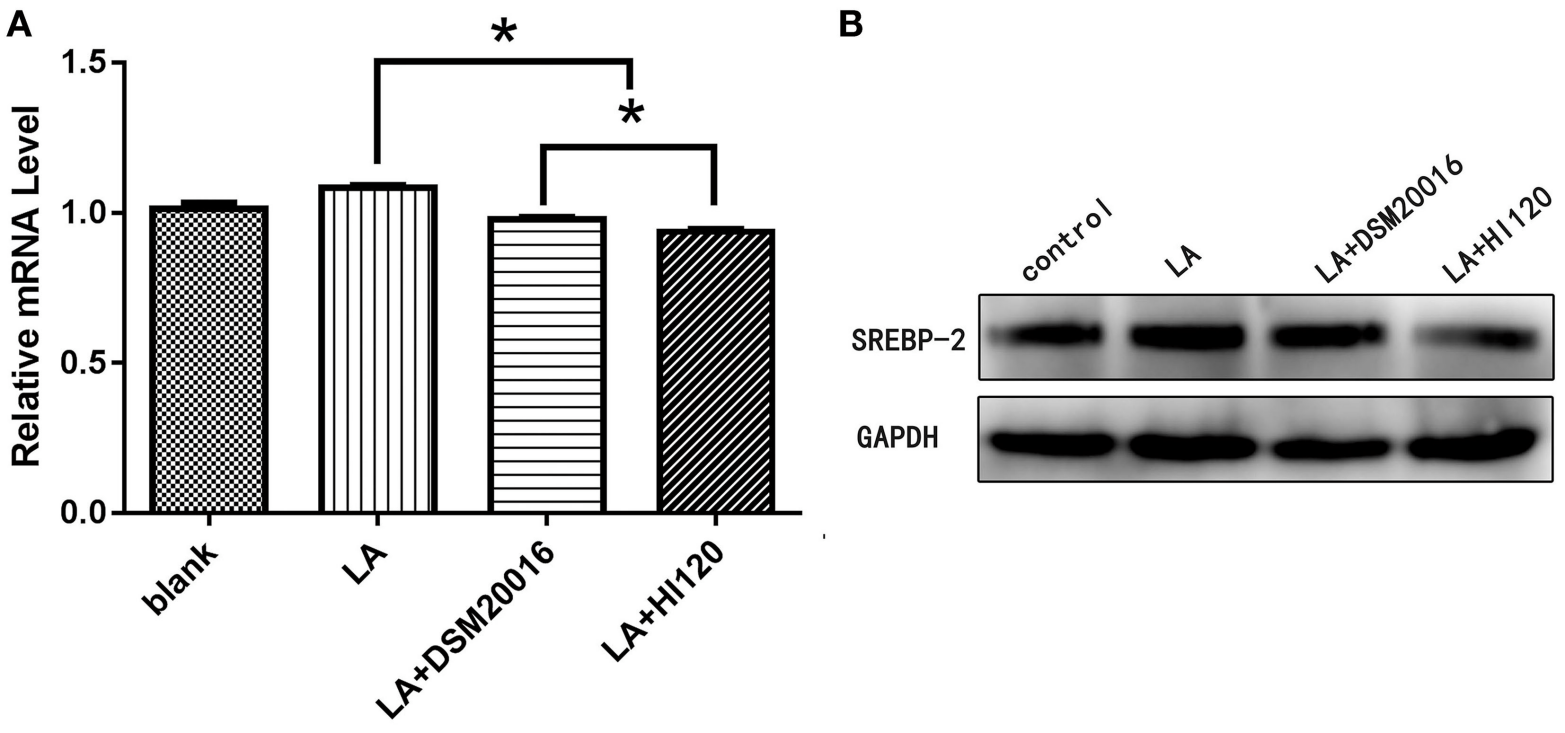
mRNA and protein expression levels of *SREBP-2* in all groups. **(A)** qRT-PCR results. **(B)** Western blotting results. *p < 0.05.

**Figure 19:**
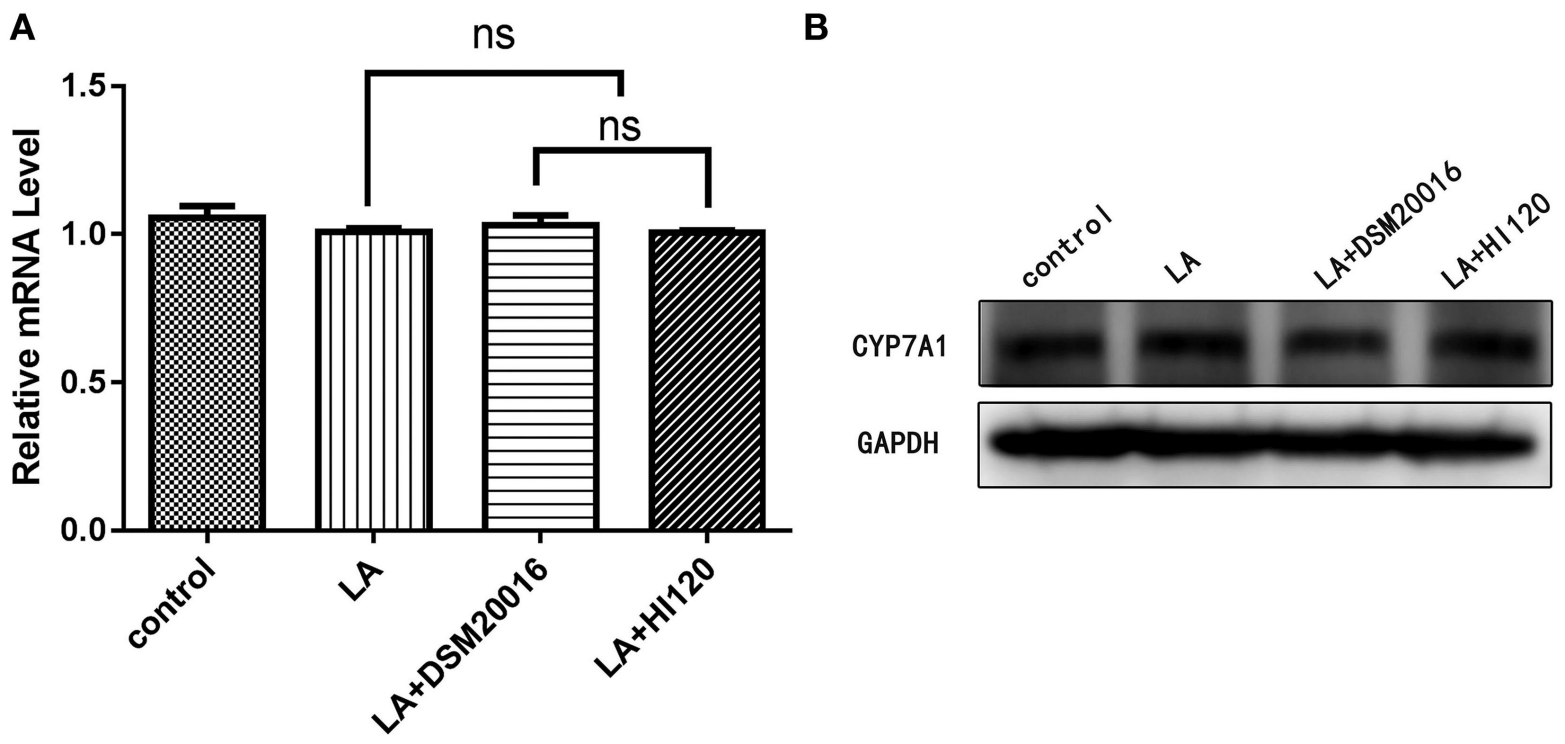
mRNA and protein expression levels of *CYP7A1* in all groups. **(A)** qRT-PCR results. **(B)** Western blotting results. *p < 0.05.

**Figure 20:**
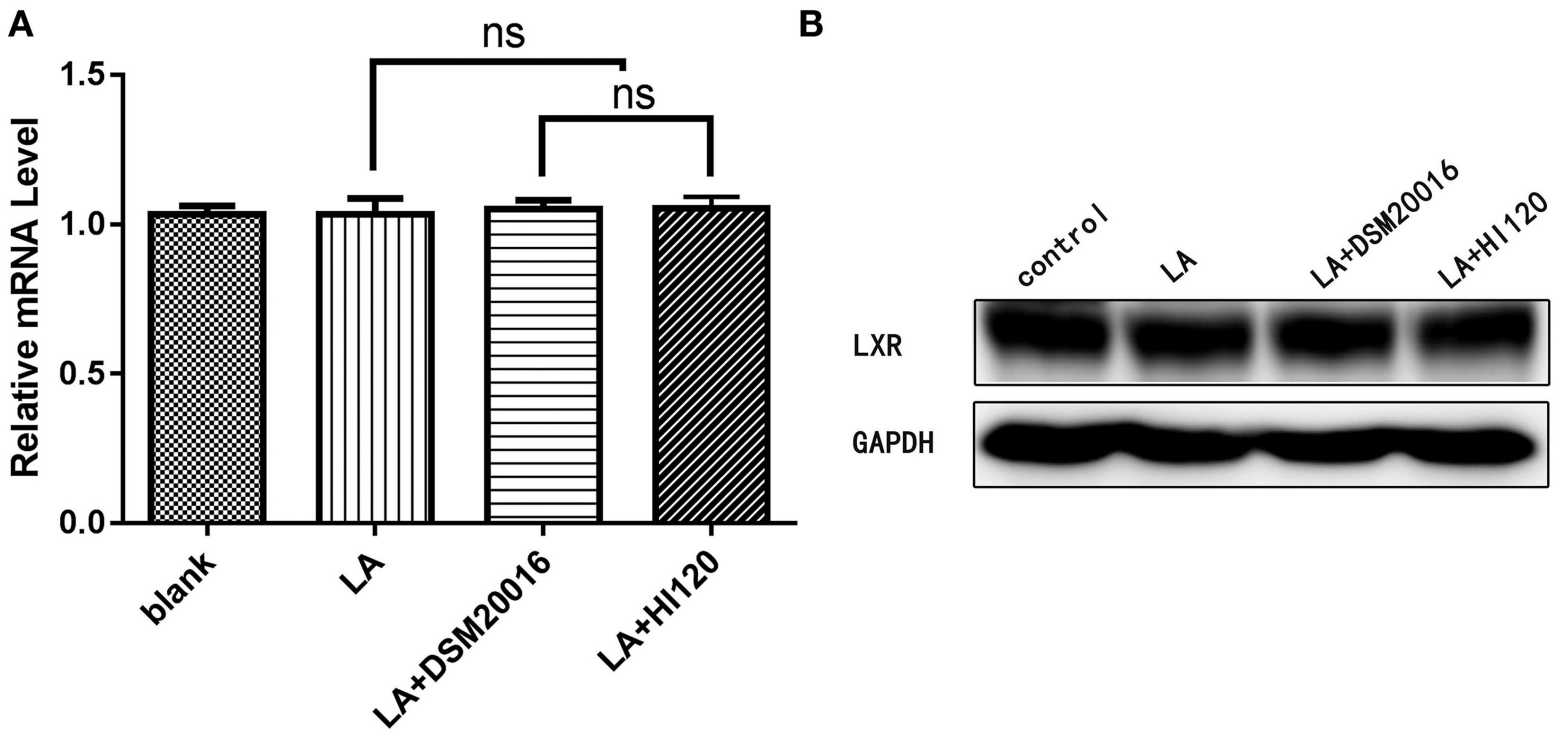
mRNA and protein expression levels of *LXR* in all groups. **(A)** qRT-PCR results. **(B)** Western blotting results. *p < 0.05.

**Figure 21:**
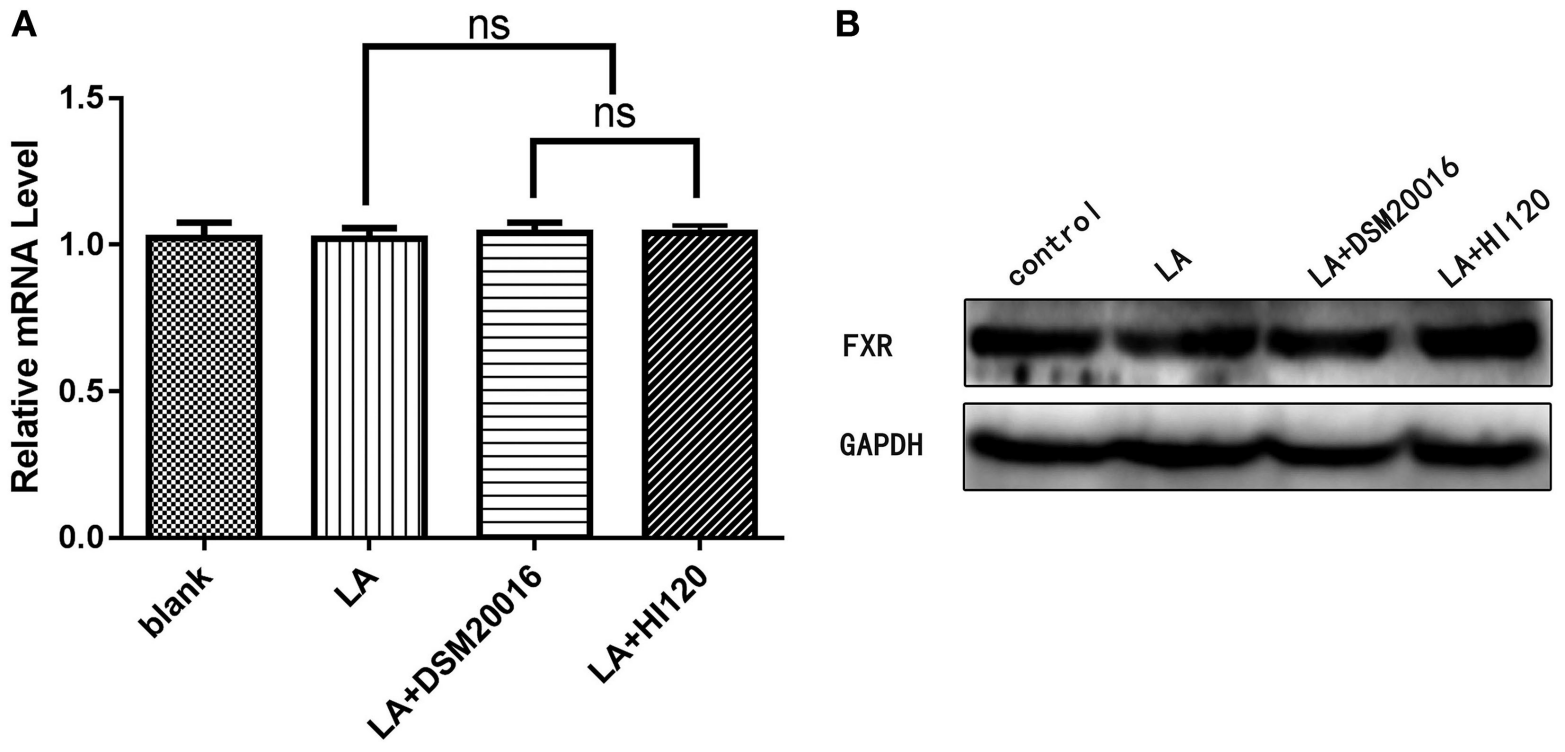
mRNA and protein expression levels of *FXR* in all groups. **(A)** qRT-PCR results. **(B)** Western blotting results. *p < 0.05.

Compared with the control group and LA group parameters, *NPC1L1* and *SREBP-2* mRNA levels and protein expression of Caco-2 cells from the HI120 group and DSM20016 group were lower; HI120 group levels were significantly lower than the corresponding DSM20016 levels (Figures 15, 16). Compared with the control group and LA group parameters, *HMG-CR* and *SREBP-2* mRNA and protein expression levels in HepG2 cells from the HI120 group and DSM20016 group were lower; HI120 group levels were significantly lower than those in the DSM20016 group (Figures 17, 18). Compared with the control group and LA group parameters, *CYP7A1, LXR*, and *FXR* mRNA and protein expression levels of HepG2 cells in the HI120 group and DSM20016 group were not significantly different; there was also no significant difference between the HI120 and DSM20016 groups (Figures 19–21).

#### NPC1L1/HMG-CR Expression After SREBP-2 siRNA Interference

**Figure 22:**
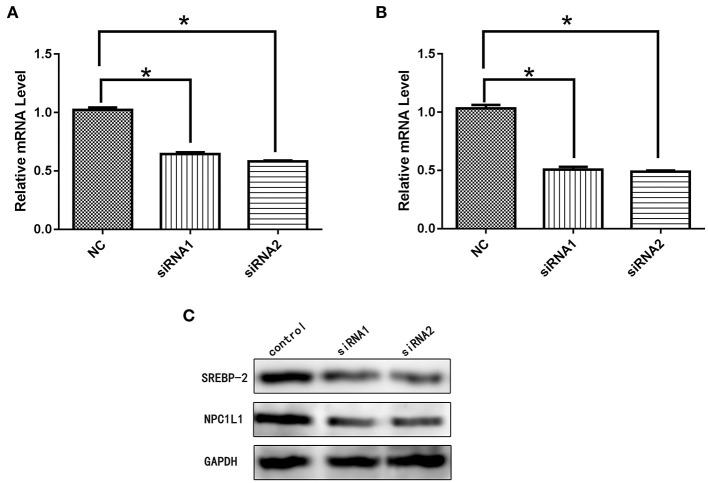
**(A)** mRNA expression level of *SREBP-2* after interference. **(B)** mRNA expression level of *NPC1L1* after *SREBP-2* interference. **(C)** *SREBP-2* and NPC1L1 protein expression levels after *SREBP-2* interference. *p < 0.05.

**Figure 23:**
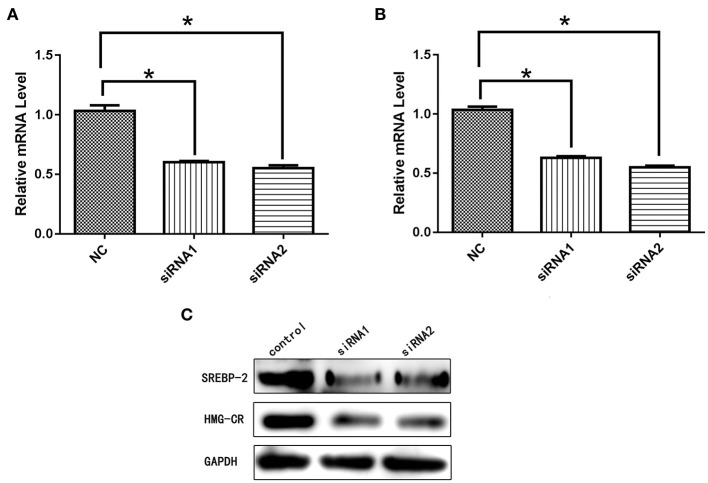
**(A)** mRNA expression level of *SREBP-2* after interference. **(B)** mRNA expression level of *HMG-CR* after *SREBP-2* interference. **(C)** *SREBP-2* and *HMG-CR* protein expression levels after *SREBP-2* interference. *p < 0.05.

After siRNA interference targeting *SREBP-2*, the mRNA and protein expression of *NPC1L1* was significantly decreased in Caco-2 cells (Figure 22). *HMG-CR* mRNA and protein expression in HepG2 cells was decreased significantly (Figure 23).

#### Immunofluorescence Detection of Cholesterol Absorption and NPC1L1/SREBP-2 Expression

**Figure 24:**
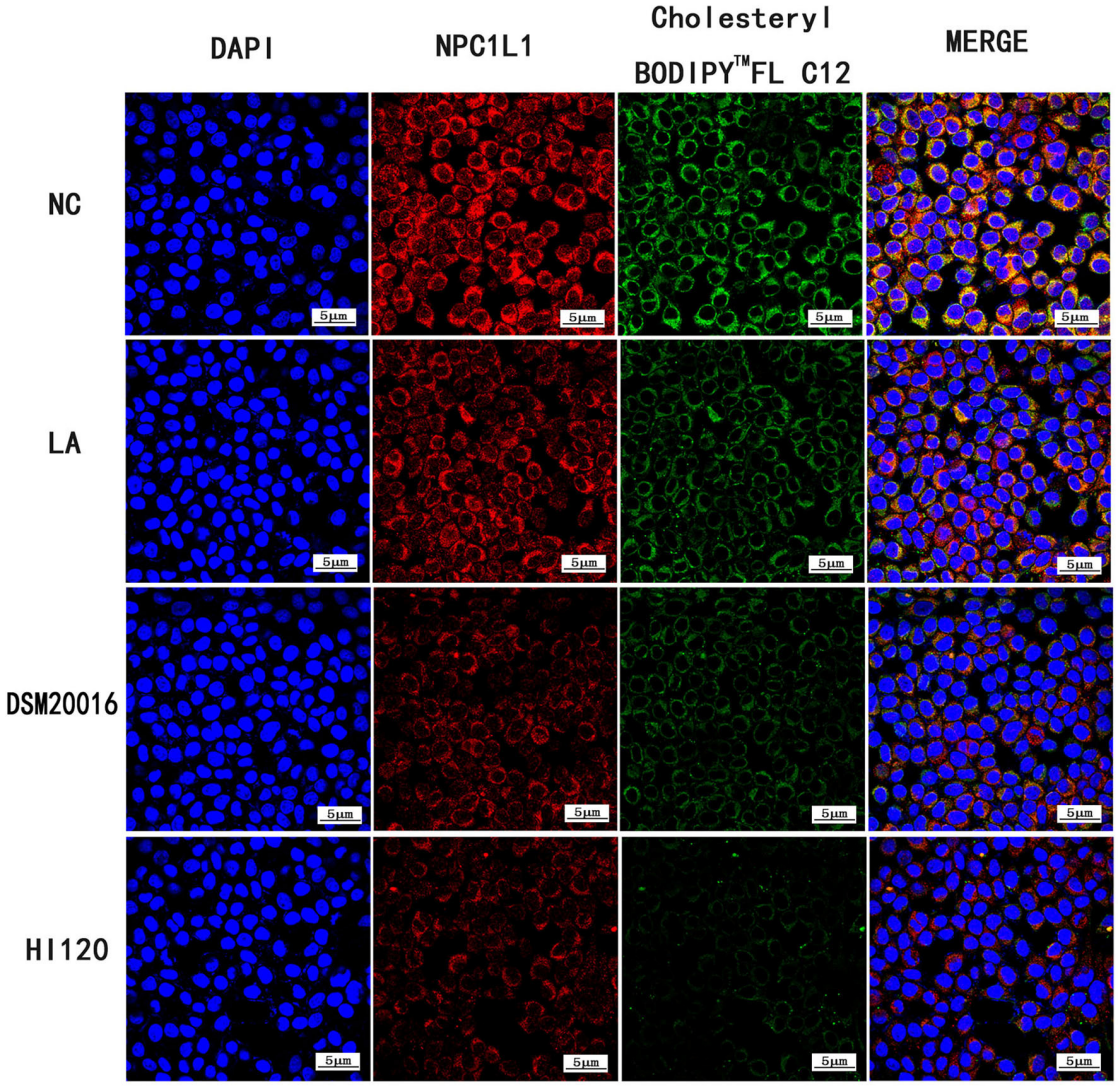
Expression of Cholesteryl Bodipy^TM^ FL C12 and *NPC1L1* observed under confocal laser microscope (600× magnification).

**Figure 25:**
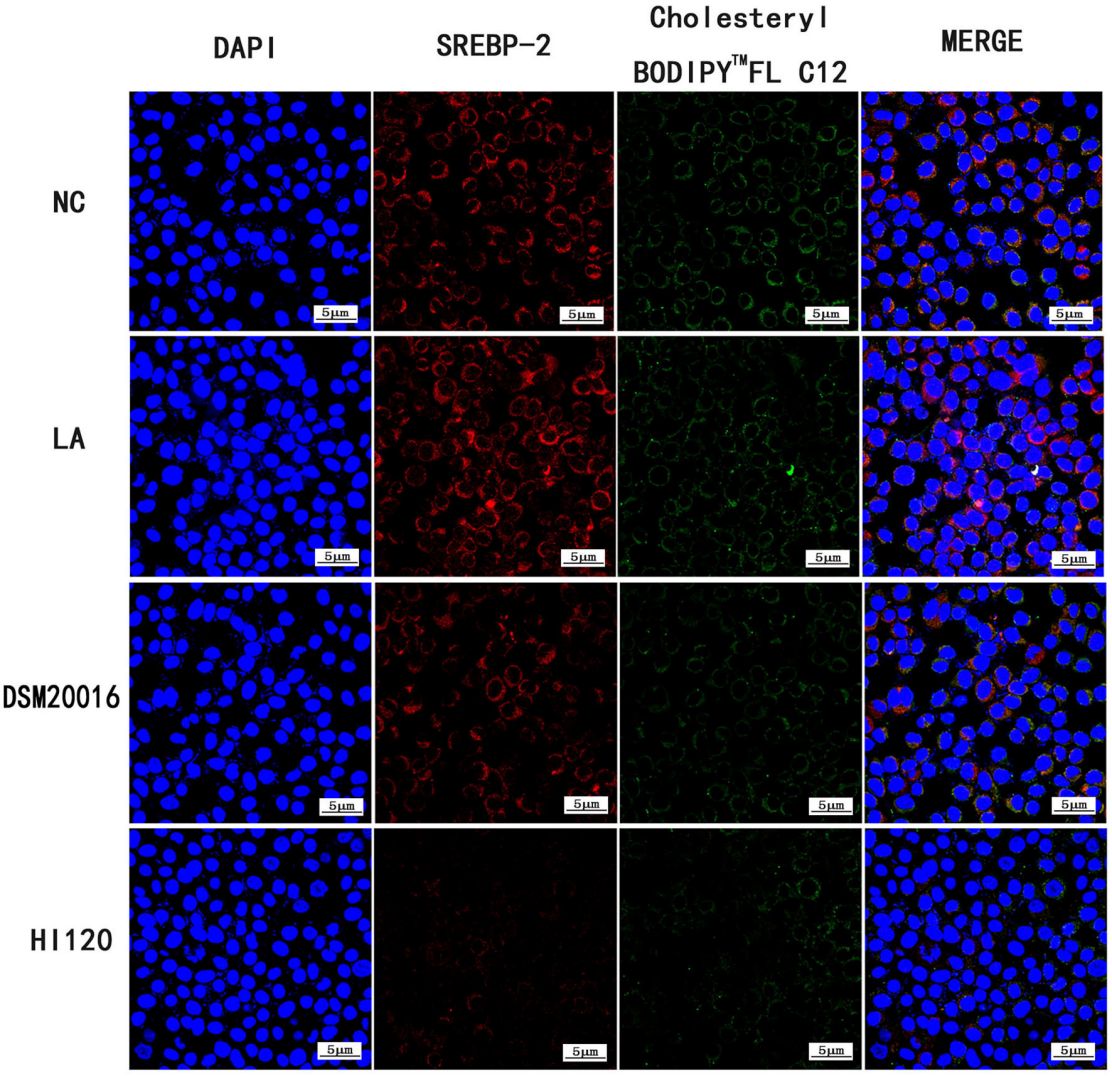
Expression of Cholesteryl Bodipy^TM^ FL C12 and *SREBP-2* observed under confocal laser microscope (600× magnification).

Compared with the NC and LA groups, the HI120 and DSM20016 groups exhibited a weak green fluorescent signal (Cholesteryl Bodipy^TM^ FL C12) and red fluorescent signal (*NPC1L1* and *SREBP-2*). The HI120 signal was weaker than that in the DSM20016 group (Figures 24, 25).

#### Fluorescence Detection of Cholesterol Absorption and NPC1L1 Expression After SREBP-2 siRNA Interference

**Figure 26:**
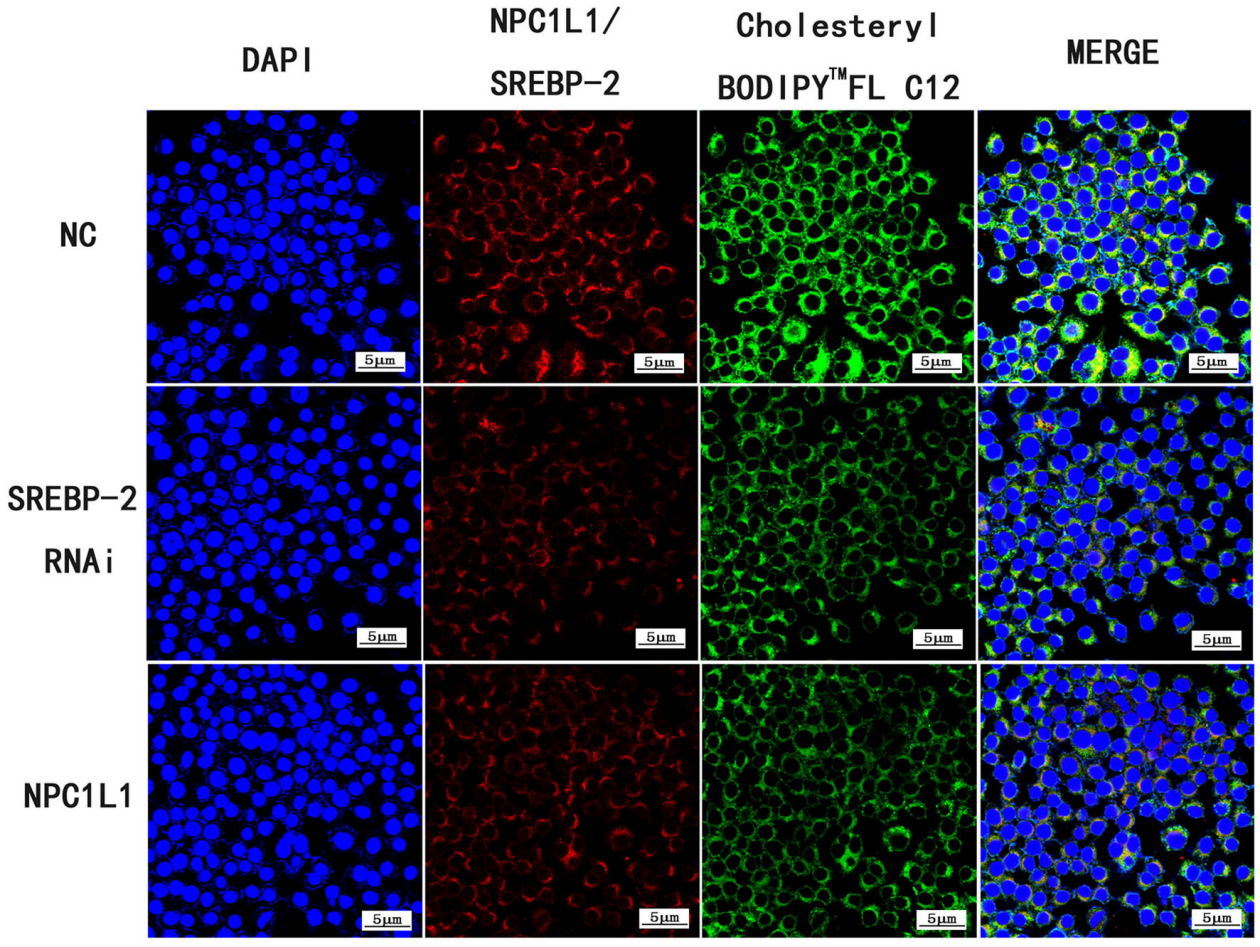
Expression of Cholesteryl Bodipy^TM^ FL C12, *SREBP-2* RNAi and *NPC1L1* under laser confocal microscope (600× magnification).

Following SREBP-2 siRNA interference, cholesterol absorption and NPC1L1 expression were decreased (Figure 26).

## Discussion

Obesity is an increasingly widespread and detrimental health condition. Among cardiovascular-related deaths, 77% are attributed to elevated cholesterol (30). For every 10% reduction in serum cholesterol, the incidence of coronary heart disease is reduced by 15% (31). The prevention and treatment of lipid metabolism abnormalities, regulation of fatty acid transport, and reduction in lipid accumulation are key for alleviating obesity, diabetes, fatty liver, and other diseases. Therefore, suitable foods and drug must be identified to reduce obesity and improve lipid metabolism.

Obesity is closely related to gut microbes (32). Some bacterial strains can reduce macrophage lipid deposition and may improve atherosclerosis (33). *Lactobacillus casei* YRL577 have the potential effect of alleviating NAFLD, and significantly reduced liver weight and liver index and could regulate the levels of lipid metabolism (34). In addition, oral administration of a strain of human FGF21 expressing *Lactobacillus* significantly reduced the weight of mice and enhanced the activity of brown adipose tissue (35).

In this study, a new *L. reuteri* HI120 was evaluated in detail, which revealed that the 16S rRNA sequence of HI120 was 99% homologous with other *L. reuteri* 16S rRNA genes, indicating that HI120 belongs to *L. reuteri*. Additionally, the LAI amino acid sequence of HI120 was 87% homologous with a *L. reuteri* standard strain. Therefore, HI120 may express high levels of LAI. After mixing *L. reuteri* HI120 with LA *in vitro*, a large amount of CLA was generated, as revealed by mass spectrometry. The produced CLA was mainly composed of C9, t11-CLA isomers.

However, unexpectedly, our *in vivo* experiments revealed no decrease in the body weight of mice fed HI120 and DSM20016. The triglyceride level of mice fed HI120 and DSM20016 also did not decrease significantly. However, the cholesterol level was decreased. This may be because the mice were given a high-fat diet for only 8 weeks, which may not have been long enough to cause large changes. However, the cholesterol level in mice fed HI120 was significantly lower than that of mice fed DSM20016, and the observed reduction was comparable to the reductions observed in the atorvastatin group. Currently, atorvastatin is the first-line treatment for reducing blood lipids, particularly cholesterol levels. Although the effect of HI120 on cholesterol reduction was as good as that of atorvastatin, this was likely related to the atorvastatin dosage. Based on these results, HI120 may be useful for preventing and treating obesity and hyperlipidemia.

To explore the causes of cholesterol reduction *in vivo*, we evaluated cholesterol absorption, synthesis, and excretion. In 2004, Altmann et al. identified the transmembrane protein NPC1L1, which is responsible for the efficient and specific transport of cholesterol into cells (22). *NPC1L1* was highly expressed in the small intestine, with highest expression in the jejunum and ileum (36). *NPC1L1* knockout resulted in a 70% reduction in cholesterol absorption efficiency, whereas the absorption of triglycerides and other lipids was not affected, indicating that NPC1L1 specifically participated in the absorption process of cholesterol in the small intestine (22). NPC1L1 is targeted by ezetimibe, the only cholesterol absorption inhibitor in use in the clinic (37). The cholesterol content of cells can regulate the transcription of *NPC1L1* via the *SREBP-2* pathway (23, 38, 39). Kumar et al. found that curcumin indirectly regulates the absorption and transport of cholesterol by *NPC1L1* by inhibiting *SREBP-2* expression (40, 41). Bisphenol A can increase cholesterol absorption of Caco-2 cells by enhancing *NPC1L1* expression, an effect that may also be regulated via *SREBP-2* (42). When Caco-2 cells were incubated with ω-3 polyunsaturated fatty acid, the expression of *NPC1L1* mRNA decreased by 35–58% (43). LA, palmitic acid, and oleic acid exerted no such effect. ω-3 polyunsaturated fatty acid regulates the absorption of cholesterol by inhibiting *NPC1L1* expression, potentially through the LXR or SREBP-2 pathway. HMG-CR is an important rate-limiting enzyme in cholesterol synthesis (24). SREBP-2 is mainly responsible for the transcriptional regulation of cholesterol synthesis genes and can maintain the relative stability of cholesterol in cells by regulating the expression of *HMG-CR* (44, 45). In mammalian cells, the *HMG-CR* mRNA level is regulated by SREBP-2. When the sterol level in cells increases, the *HMG-CR* mRNA level decreases significantly. When the sterol level is low, *SREBP-2* expression is induced, resulting in transcription of *HMG-CR* (24). CYP7A1 is the key and rate-limiting enzyme of bile acid production (cholesterol excretion). By interfering with *CYP7A1* expression and reducing the synthesis of bile acids in mice, the absorption efficiency of cholesterol can be significantly reduced (25). The positive regulatory factor of *CYP7A1* is LXR, whereas FXR is the negative regulatory factor. LXR and FXR cooperate to control the level of bile acids (46). Bile acid feedback regulation is mediated via the transcription regulation of *CYP7A1* by FXR protein (26). LXR can promote *in vivo* reverse cholesterol transport and upregulate *CYP7A1* (27, 47, 48).

The current study examined the expression of genes related to cholesterol synthesis, absorption, and metabolism. *In vivo* and *in vitro* experiments revealed that the expression of *NPC1L1, SREBP-2*, and *HMG-CR* was decreased, whereas that of *CYP7A1, FXR*, and *LXR* was not changed significantly by probiotic treatment. CLA, generated by two *L. reuteri* strains, reduced the gene expression of *SREBP-2* and *NPC1L1*, contributing to reduced cholesterol absorption. Furthermore, *SREBP-*2 and *HMG-CR* expression was reduced, potentially leading to lower cholesterol synthesis. HI120 exhibited a more prominent effect than DSM20016. However, whether cholesterol levels are affected via corresponding metabolic pathway remains unclear. We used BODIPY fluorescence (49) to label cholesterol to assess the molecular mechanism of absorption. The results revealed that CLA regulates NPC1L1 and cholesterol absorption through SREBP-2. By siRNA-mediated interference of *SREBP-2, SREBP-2* was confirmed to regulate *NPC1L1* and *HMG-CR* and thus affect the absorption and synthesis of cholesterol.

CLA of high purity is costly, and industrially produced CLA cannot be used in the daily diet, as food safety cannot be guaranteed. The edible oil in people's daily diet contains a considerable amount of LA. Long-term use of *L. reuteri*, a safe and reliable edible strain, may contribute to cholesterol reduction, supporting the prevention and treatment of high blood lipid–related disease. It is necessary to consider that the dosage used in the present study and duration of the experiments may need to be adjusted. Further investigation of whether HI120 can directly reduce cholesterol and the underlying mechanism is needed.

## Data Availability Statement

All datasets generated for this study are included in the article/supplementary material.

## Ethics Statement

The animal study was reviewed and approved by Institutional Laboratory Animal Care and Use Committee of the South Medical University at Guangzhou.

## Author Contributions

YS performed the experiments with bacteria, mice, cells, analyzed the data, and wrote the manuscript. XH and YT performed the experiments with mice. HW and LH performed the experiments with bacteria. JW provided the bacteria. HN and WZ designed the experiments, analyzed the data, and contributed to revising the manuscript. YB provided overall direction and contributed to revising the manuscript. All authors contributed to the article and approved the submitted version.

## Conflict of Interest

JW was employed by the Guangzhou Weisengene Biological Technology Co., Ltd. The remaining authors declare that the research was conducted in the absence of any commercial or financial relationships that could be construed as a potential conflict of interest.
